# The deubiquitinase USP7 and E3 ligase TRIM21 regulate vasculogenic mimicry and malignant progression of RMS by balancing SNAI2 homeostasis

**DOI:** 10.1186/s13046-024-03056-1

**Published:** 2024-05-04

**Authors:** Ruyue Zhang, Daidi Zhang, Fugen Han, Xiaorui Song, Yaodong Zhang, Jie Zhang, Qingwen Zhu, Yanru Qin

**Affiliations:** 1 Department of Clinical Oncology, The First Affiliated Hospital of Zhengzhou University, Zhengzhou, 450052 China; 2 Department of Otorhinolaryngology Head and Neck surgery, Children’s Hospital Affiliated to Zhengzhou University, Zhengzhou, 450052 China; 3 Henan Province Engineering Research Center of Diagnosis and Treatment of Pediatric Infection and Critical Care, Children’s Hospital Affiliated to Zhengzhou University, Zhengzhou, 450052 China; 4 Department of Otorhinolaryngology Head and Neck surgery, National Center for Children’s Health, Beijing Children’s Hospital Capital Medical University, Beijing, 10045 China; 5 Department of Otorhinolaryngology Head and Neck Surgery, The First Affiliated Hospital of Zhengzhou University, Zhengzhou, 450052 China

**Keywords:** Rhabdomyosarcoma, Vasculogenic mimicry, Deubiquitination, Ubiquitination, SNAI2, USP7, TRIM21

## Abstract

**Background:**

Rhabdomyosarcoma (RMS) is a rare malignancy and the most common soft tissue sarcoma in children. Vasculogenic mimicry (VM) is a novel tumor microcirculation model different from traditional tumor angiogenesis, which does not rely on endothelial cells to provide sufficient blood supply for tumor growth. In recent years, VM has been confirmed to be closely associated with tumor progression. However, the ability of RMS to form VM has not yet been reported.

**Methods:**

Immunohistochemistry, RT-qPCR and western blot were used to test the expression level of SNAI2 and its clinical significance. The biological function in regulating vasculogenic mimicry and malignant progression of SNAI2 was examined both in vitro and in vivo. Mass spectrometry, co-immunohistochemistry, immunofluorescence staining, and ubiquitin assays were performed to explore the regulatory mechanism of SNAI2.

**Results:**

Our study indicated that SNAI2 was abnormally expressed in patients with RMS and RMS cell lines and promoted the proliferation and metastasis of RMS. Through cell tubule formation experiments, nude mice Matrigel plug experiments, and immunohistochemistry (IHC), we confirmed that RMS can form VM and that SNAI2 promotes the formation of VM. Due to SNAI2 is a transcription factor that is not easily drugged, we used Co-IP combined with mass spectrometry to screen for the SNAI2-binding protein USP7 and TRIM21. USP7 depletion inhibited RMS VM formation, proliferation and metastasis by promoting SNAI2 degradation. We further demonstrated that TRIM21 is expressed at low levels in human RMS tissues and inhibits VM in RMS cells. TRIM21 promotes SNAI2 protein degradation through ubiquitination in the RMS. The deubiquitinase USP7 and E3 ligase TRIM21 function in an antagonistic rather than competitive mode and play a key role in controlling the stability of SNAI2 to determine the VM formation and progression of RMS.

**Conclusion:**

Our findings reveal a previously unknown mechanism by which USP7 and TRIM21 balance the level of SNAI2 ubiquitination, determining RMS vasculogenic mimicry, proliferation, and migration. This new mechanism may provide new targeted therapies to inhibit the development of RMS by restoring TRIM21 expression or inhibiting USP7 expression in RMS patients with high SNAI2 protein levels.

**Supplementary Information:**

The online version contains supplementary material available at 10.1186/s13046-024-03056-1.

## Introduction

Rhabdomyosarcoma (RMS) is the most frequent type of soft tissue sarcoma in children, representing approximately 4.5% of all pediatric cancers [1]. Despite advancements in pediatric cancer treatment, patients with RMS have a bleak prognosis, with a survival rate of less than 30% in 5 years due to its rapid progression, recurrence, and metastasis [2]. Thus, investigations into the molecular mechanisms that drive RMS proliferation and metastasis are crucial to inform RMS treatment and prognostic assessment.

Hematogenous metastasis is one of the primary pathways of tumor metastasis. In 1971, Professor Judah Folkman first proposed the concept of “anti-angiogenesis”, positing that solid tumors heavily depend on the sprouting of new capillaries. Without neovascularization, most solid tumors cease to grow beyond 2–3 mm in size and enter a dormant state [3]. However, it is regrettable that in certain highly malignant tumors, “anti-angiogenic drugs” have far from achieved the anticipated outcomes [4–7]. Some researchers have hypothesized that the poor efficacy of anti-angiogenic treatment might be due to the existence of alternative blood supply mechanisms in tumors. In 1999, Maniotis et al. discovered that melanoma cells could differentiate into cells with endothelial-like characteristics and form tubular structures [8]. However, these structures do not contain endothelial cells, yet the lumens carry red blood cells and can perform the same function as traditional blood vessels. Therefore, the formation of such structures is termed vasculogenic mimicry (VM). VM is a unique model of tumor microcirculation that differs from traditional tumor angiogenesis in that it does not depend on endothelial cells to supply sufficient blood for tumor growth [9]. Clinically, VM is usually assessed in patient samples by identifying vessels that are positive for periodic acid-Schiff (PAS) staining but negative for CD34, as indicated by IHC analysis. PAS stains the basement membrane, whereas CD34 are endothelial cell markers [10]. VE-cadherin was one of the initial molecules that stimulated VM in aggressive melanoma and is the primary indicator of VM [11]. In further research, VM has been identified not just in uveal melanoma but also in liver cancer, nasopharyngeal carcinoma, prostate cancer, and ovarian cancer [12–19]. Furthermore, VM is significantly linked to tumor growth, differentiation, invasiveness, and an unfavorable prognosis in patients affected by malignant tumors [20–24]. VMs comprise cancer cells, and their channel formation mechanism differs from that of blood vessels formed by endothelial cells, explaining why VEGF-targeted therapy is suboptimal in its efficacy.

To date, insufficient research has been conducted on VM establishment in RMS. However, our earlier study using a cell-derived xenograft (CDX) model in nude mice found VM present in RMS tissue. Moreover, subsequent in vitro experiments revealed that human RMS cells (RD, A204) can create tubules similar to those of human vascular endothelial cells (HUVEC) via tubular formation (Fig. S1A). In addition, experiments using matrix plugs in nude mice demonstrated that RMS cells (RD, A204) possess CD34-/PAS + characteristics, contrasting with the CD34 + /PAS + features found in HUVECs (Fig. S1B). Collectively, these experiments show that RMS cells can form VM both in vitro and in vivo. Thus, it is crucial to investigate human RMS samples to confirm the presence of vasculogenic mimicry.

SNAI2, a member of the SNAIL zinc finger transcription factor superfamily, serves as an epithelial-mesenchymal transition (EMT) transcription factor [25, 26]. Several recent studies have shown that SNAI2 shows high expression levels in primary malignant tumors like breast, pancreatic, and gastric cancers [27–29]. It plays a crucial role in regulating the behavior of malignant tumors through EMT and non-EMT pathways. Abnormal expression of SNAI2 in tumor tissue correlates with low differentiation, recurrence, metastasis, and poor prognosis [30–32]. However, limited studies explore the role of SNAI2 in the malignant progression of RMS.

Ubiquitin-specific protease 7 (USP7) is an extensively researched deubiquitination enzyme (DUB) [33]. Its enzymatic activity influences the localization, activation, and stability of its substrates [34, 35]. Numerous studies have indicated higher expression of USP7 in tumors, including breast, cervical, and colorectal cancers [36–38]. Research has shown that USP20, a deubiquitinase, promotes breast cancer metastasis by stabilizing SNAI2 [39]. Nevertheless, the relationship between USP7 and SNAI2 in RMS remains to be explored.

The TRIM protein family comprises 77 highly conserved E3 ligases, which are regarded as critical regulators of protein degradation via ubiquitination [40]. Most members possess a ringed zinc-finger domain, one or two B-box domains, along with a curly helix domain, and a variable C-terminal domain [41]. The TRIM protein family participates in several cellular physiological processes, and an unevenness in its protein content could cause tumorigenesis [42–45]. However, the precise role of TRIM21 in RMS progression remains poorly understood.

The study aims to investigate the role of SNAI2 in the malignant progression of RMS and its association with VM. Our study found that the expression of SNAI2 is increased in RMS tissues and is associated with clinical features such as tumor stage and metastasis. Furthermore, SNAI2 contributes to the malignant biological progression of RMS by promoting proliferation, migration, and VM. Subsequently, we identified the SNAI2-binding protein USP7 and will further explore the interaction between USP7 and SNAI2 in RMS and their effects on VM and malignant progression. With further investigation, we discovered the E3 ligase TRIM21 that may bind to SNAI2. Based on the above results, we plan to conduct experiments to explore whether USP7 and TRIM21 exert antagonistic regulation on the stability of SNAI2, thus affecting VM, proliferation and migration of RMS. This will help identify potential therapeutic targets and new biological markers for RMS.

## Materials and methods

### Human RMS specimens

With ethics committee approval, we collected 59 tumor tissues and 12 normal muscle tissues of RMS from the Children's Hospital affiliated with Zhengzhou University. All patients provided informed consent and underwent no cancer treatment before the biopsy. Tissue samples were stored at -80 °C until use. IHC was used to detect the expression of SNAI2, USP7, TRIM21, CD34/PAS, and VE-cadherin.

### Cell culture

Human RMS cell lines (RD, A204, RH30, and A673), HSkMCs, and HUVECs were purchased from Wuhan Punoxai Biotechnology Co., Ltd. RMS cells were cultured in DMEM supplemented with 10% fetal bovine serum (FBS; Thermo Fisher Scientific Corporation). HSkMCs and HUVECs cells were cultured in DMEM/F-12. All cell lines were identified by short tandem repeat (STR) fingerprinting and tested negative for Mycoplasma contamination before the experiment.

### Immunohistochemistry (IHC)

Immunohistochemistry was performed as previously described [46]. Staining intensity grades were divided into levels 0 (negative), 1 (weakly positive), 2 (positive), and 3 (strongly positive), and the dyeing area score was divided into 1 (0%–25%), 2 (26%–50%), 3 (51%–75%), and 4 (> 75%). The final staining score was defined as the product of the two scores, with a score of 0–6 defined as low expression and a score of 7–12 as high expression.

### Transfection with plasmids and lentiviral vectors

All transfection experiments were performed as previously described [47]. Plasmids shSNAI2, shUSP7 and shTRIM21 and their lentiviral packaging were commissioned for Genepharma (Shanghai, China) synthesis, and overexpressed plasmids (FLAG-tagged SNAI2, FLAG- or HA- or Myc-tagged USP7/wt, Myc-tagged USP7/C223S, HA-Ub/mt, HA-Ub/wt, Myc-tagged TRIM21) and their lentiviral packaging were commissioned for OBiO (Shanghai, China). siUSP7 and negative controls were purchased from GenePharma. RD and A204 cells were infected with leniviruses, and stable cells were screened with 2ug puromycin. The shRNA sequences used were as follows: shNC:5’-GTTCTCCGAACGTGTCACGT-3’;shSNAI2:5’-CCAGAATGTCTCTCCTGC-3’;shUSP7:5’-CCTGGATTTGTGGTTACGTTA-3’;shTRIM21:5’-TGAGAAGTTGGAAGTGGAAAT-3’; siNC: 5’-UUCUCCGAACGUGUCACGU-3’; USP7 siRNA-1:5’-ACCCUUGGACAAUAUUCCUTT-3’;USP7 siRNA-2: 5’-AGUCGUUCAGUCGUCGUAU-3’.

### Antibodies and reagents

The antibodies used for western blotting (WB), Co-IP, IHC and immunofluorescence (IF) in this study included antiSNAI2 antibody (9585, CST, USA), anti-USP7 antibody (66,514–1-lg, Proteintech, China), anti-TRIM21 antibody (12,108–1-AP, Proteintech, China), anti-CD34 antibody (11,265–1-AP, Proteintech, China), anti-Flag antibody (66,008–4-Ig, Proteintech, China), anti-Myc antibody (16,286–1-AP, Proteintech, China), anti-HA antibody (51,064–2-AP, Proteintech, China), mouse IgG (3420, CST),,protein A/G agarose beads, anti-Flag Magnetic Beads, anti-Myc Magnetic Beads, anti-HA Magnetic Beads (Beyotime, China) and MG132 (C2211, Sigma Aldrich) was dissolved in dimethyl sulfoxide.

### qRT‑PCR

Total RNA was isolated from cultured cells with TRIzol Reagent (Invitrogen, USA). Specifc primers and Power SYBR Green PCR Master Mix (Applied Biosystems) were used to amplify cDNA after reverse transcription of RNA samples by 5 × HiScript®II qRT SuperMix (Vazyme Biotech, China). RNA expression levels were normalized to β-actin expression. The specifc primers for SNAI2 were as follows: 5’-TGTTGCAGTGAGGGCAAGAA-3’(sense), 5’-GACCCTGGTTGCTTCAAGGA -3’(antisense); for.

USP7, 5’- GTCACGATGACGACCTGTCTGT -3’ (sense),

5’- GTAATCGCTCCACCAACTGCTG -3’ (antisense);

For TRIM21, 5’- CAGAACTCAGGAGTGTGTGCCA -3’.

(sense), 5’-TCCAAGCCTCACTTGTCTCCGA-3’ (antisense); for β-actin, 5’- CCTGGCACCCAGCACAAT -3’ (sense), 5’- GGGCCGGACTCGTCATAC -3’ (antisense).

### Western blotting

A total protein extraction buffer (GLPBIO, Montclair, CA, USA, #GK10023) containing protease inhibitors (GLPBIO #10,014) and phosphatase inhibitors (GLPBIO 152#GK10013) was used to extract total protein. In order to separate protein samples, sodium dodecyl sulfate–polyacrylamide gel electrophoresis was used PVDF membranes (Millipore, Billerica, MA, USA) as a transfer medium. 5% BSA was used to block membranes for 2 h and incubated them with primary and secondary antibodies. Blots were visualized with a Bio-Rad 1,708,265 image system (Bio-Rad, USA), using β-actin or GAPDH as a control.

### Co-IP and mass spectrometry

Immunoprecipitation assay was performed as described in Lv et al. [48]. Cells were lysed in the pre-cooled non-denatured lysis solution (lysis buffer mixed with protease inhibitor cocktail in a ratio of 100:1). The protein lysate (5%) was analyzed by western blot analysis (input). Then the remaining lysate was added to protein A/G agarose beads pre-coupled with antibody and incubated at 4 °C for 4 h. The beads were washed and the differentially protein bands were analyzed by mass spectrometry (Biotprofile, Shanghai, China), then boiled in 2 × SDS loading buffer for WB analysis.

### Cell counting kit (CCK-8) and colony formation assays

For the cell viability assay, cells were seeded into 96-well plates. At specific time points (0, 1, 2, 3 days), 10 µl of CCK-8 (DOJINDO, Japan) was added to each well, and the absorbance at 450 nm was measured using a spectrophotometer 2 h later. For the clone formation assay, cells were seeded into 6-well plates. After 7 days, the plates were washed, fixed, and stained, and the colonies were counted.

### Wound healing and transwell assays

The migratory ability of cells was evaluated using a wound healing assay. A six-well plate was seeded with cells. The cells were wound with a 10 ul pipette tip after reaching 100% confluence, and then cultured in basic medium in an incubator using basic medium. At specific times, the wound area was quantified using ImageJ software. Each experiment was repeated thrice. There were two types of transwell assays: migration and invasion. Using Transwell inserts (Corning, #3422) with an 8 µm pore size, we added 4 × 10^4^ cells resuspended in basic medium to the upper chamber, and 10% foetal bovine serum to the lower chamber. Transwell invasion assays involved the use of prepared 60 µl matrigel (Corning, #356,234) pre-coated onto the upper membrane. Cells were fixed with 4% paraformaldehyde and stained with 0.1% crystal violet (Solarbio, China) following an 18 h migration assay or 36 h invasion assay. Digital images of the membranes were obtained by photographing three random fields in each chamber.

### Matrigel tube formation assay

Matrigel tube formation assay was conducted to evaluate the ability of RMS cells to form vessel-like structures, which is a key event in vasculogenic mimicry. After dissolving the Matrigel at 4 °C, 60 μl of Matrigel was added to each well of a 96 well plate, and allowed to polymerize at 37 °C for 1 h. Subsequently, 4 × 10^4 RMS cells were resuspended in 100 μl of DMEM/F12 medium and incubated at 37 °C in 5% CO2. The tube formation of the cells was observed every 6 h.

### Immunofuorescence

Cells were seeded into 10 mm glass dishes coated with with 4% paraformaldehyde and 5% BSA at room temperature for 30 min. Fixed cells were incubated overnight at 4 °C with primary antibodies against VE-cadherin (1:200), SNAI2 (1:50), USP7 (1:50), TRIM21 (1:50).

### In vivo ubiquitination assays

RD Cells were transfected with HA-Ubiquitin and indicated plasmids for in vivo ubiquitination assay, which was performed as previously described [49].

### Zebrafish xenograft model

Zebrafish xenograft models were established following previous procedures [50]. The zebrafish were bred and kept at the Pediatric Research Institute of the Children's Hospital Affiliated to Zhengzhou University. Tumor cells underwent two rounds of PBS washing and were then labeled with DiI (Beyotime, #C1036) at a concentration of 5 g/mL. After 48 h of fertilization, the zebrafish were anesthetized with 1.2 mM tricaine and placed in a modified agarose gel mold for microinjection of tumor cells. Tumor cells (300) were injected into the perivitelline cavity of zebrafish embryos using a 5 nL DMEM medium. On day 5 after injection, the zebrafish xenograft specimens were fixed with neutral formalin, embedded in paraffin, and sectioned at a thickness of 10 μm. Using standard methods, the sections were stained with hematoxylin (HE) and observed under an optical microscope.

### BALB/c nude mice models

#### Matrigel plug vascular mimicry assay

A mixture of 200 μL cells and Matrigel (5 × 10^6^ cells/mouse) was injected subcutaneously into 5-week-old male nude mice. One week later, the specimens were collected, fixed, sectioned, and immunohistochemically stained.

### Cell-derived xenograft (CDX)

Four-week-old nude mice were injected subcutaneously with stably transfected RD cells (5 × 10^6^ cells/mouse). After 4 weeks, the tumors were collected, fixed, sectioned, and stained.

### Lung metastasis in the nude mouse model

First, cell suspensions of four groups of RD cells (shNC, shUSP7Z, shTRIM21, and shTRIM21 + shUSP7) were prepared at a density of 4 × 10^7^ cells/mL and temporarily stored on ice. The mice were fixed, and their tails were wiped with alcohol cotton balls to fill the tail blood vessels before injection. Each nude mouse was injected with 100 µL single-cell suspension. The mice were euthanized four weeks later and dissected to observe lung metastasis.

### Statistical analysis

The study used Pearson's test with R software for correlation analysis. Results are presented as mean ± standard deviation (SD) from three independent experiments. All statistical analyses were performed using GraphPad Prism 5.2 software, including the student's t-test for two-group comparisons and one-way analysis of variance for multiple group comparisons. Each experiment was repeated independently at least three times. Asterisks (*, **, *** and ****) stand for *P* < 0.05, *P* < 0.01, *P* < 0.001, and *P* < 0.0001, respectively.

## Results

### SNAI2 is highly expressed in RMS and associated with clinical features

**Fig. 1:**
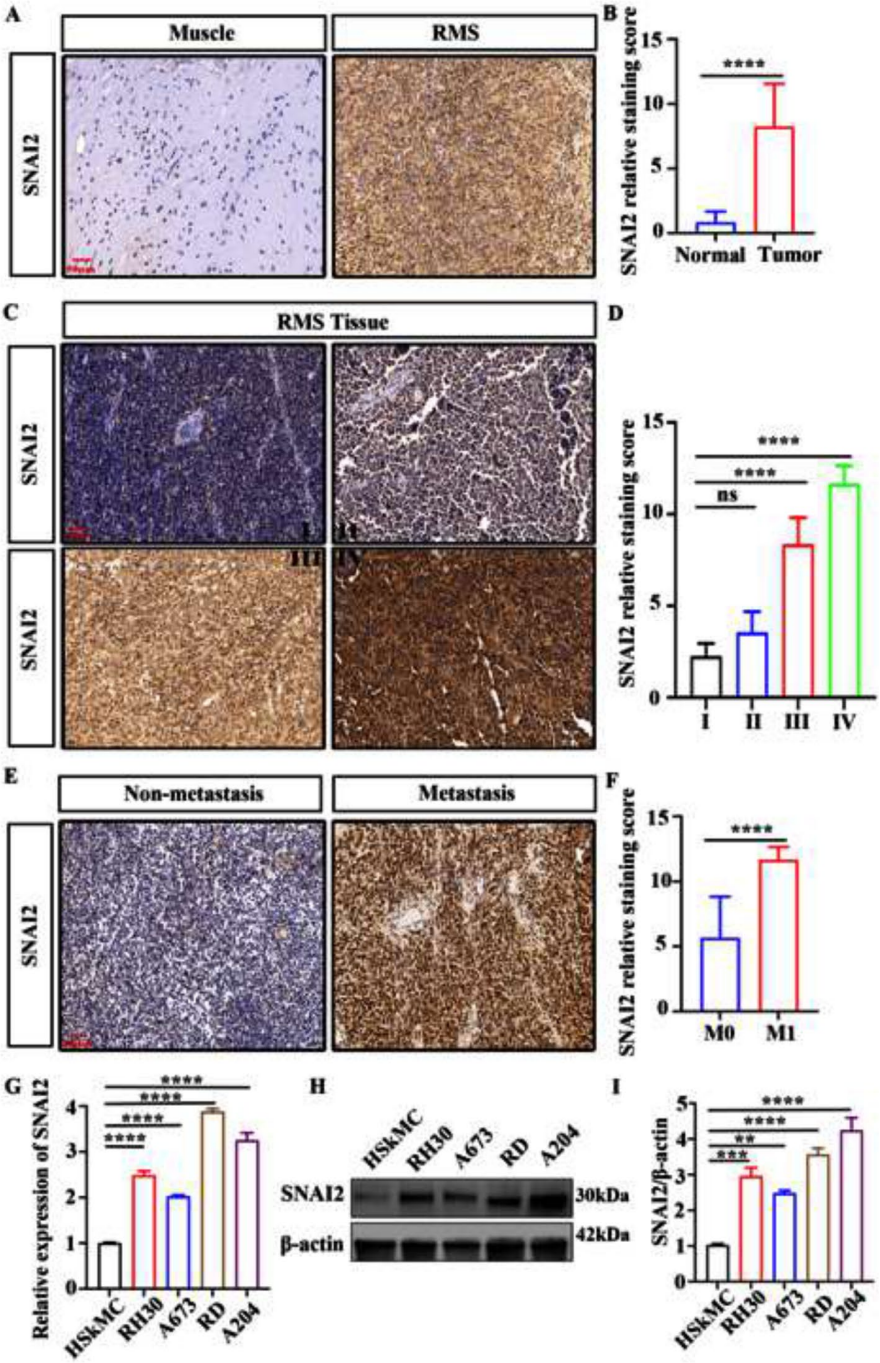
The SNAI2 expression in human RMS tissues and its correlation with clinical characteristics. **A**, **B** The expression level of SNAI2 in 59 RMS tissues and 12 normal tissues. **C**, **D** The expression of SNAI2 is associated with tumor stage in RMS patients. **E**, **F** The expression level of SNAI2 in metastasis tissues and no-metastasis tissues. **G** The expression of SNAI2 mRNA was analyzed by RT-PCR in RMS cells and normal skeletal muscle cell lines. **H**, **I** Western blotting analysis and quantification of SNAI2 protein levels in RMS cells and normal skeletal muscle cell lines. Data are presented as the Mean ± SD. ^***^P < 0.001 and^****^P < 0.0001

This study analyzed the expression of SNAI2 in 34 primary RMS samples, 25 metastatic tumor samples, and 12 normal muscle tissue samples collected from Zhengzhou University Children's Hospital between 2010 and 2022. Findings showed that SNAI2 expression levels in RMS tissues were significantly higher than in normal muscle tissues (Fig. 1A, B). Furthermore, patients with stage III-IV disease displayed higher SNAI2 expression levels compared to those with stage I-II disease (Fig. 1C, D). SNAI2 expression was significantly increased in metastatic patients as opposed to non-metastatic patients (Fig. 1E and F). Moreover, mRNA and protein expression of SNAI2 were upregulated in four RMS cell lines (RH30, A673, RD and A204) compared to normal human skeletal muscle cell lines (HSkMCs) (Fig. 1G-I).

### SNAI2 contributes to the malignant biological progression of RMS in vitro and in vivo

#### In vitro

**Fig. 2:**
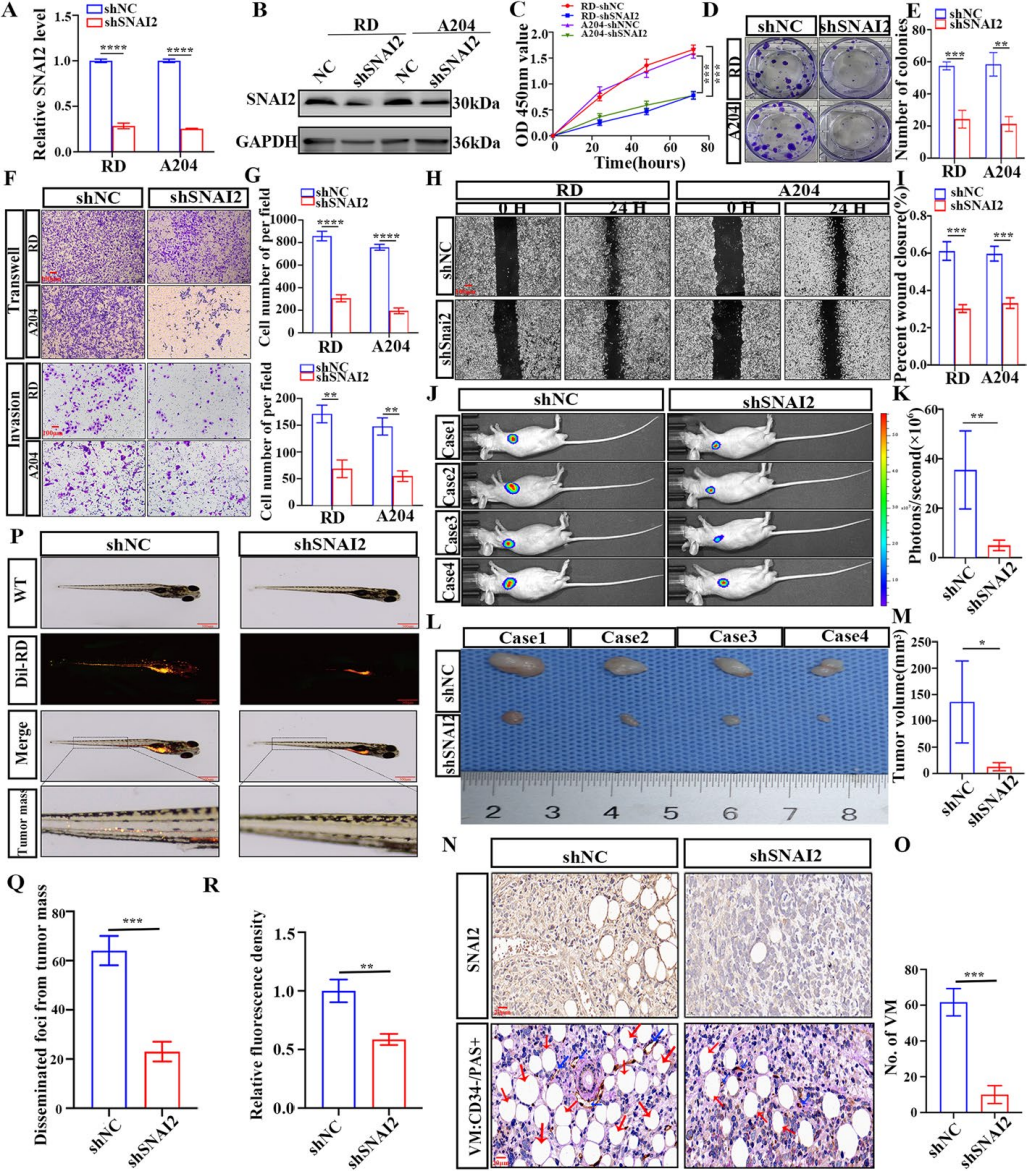
SNAI2 contributes to the malignant progression of RMS in vitro and in vivo. **A** The expression of SNAI2 mRNA was analyzed by RT-PCR in RMS cells infected with shRNAs. **B** Western blotting analysis of SNAI2 protein level in RMS cells infected with shRNAs. **C** CCK8 assay in RMS cells transfected with shSNAI2. **D**, **E** Colony formation of RMS cells transfected with shSNAI2 (**D**). The colony counts were normalized to the control and expressed as a percentage, and results are represented in the bar graph (**E**). **F**, **G** Representative images (**F**) and Graphic representation (**G**) of the migration and invasion capacities in the SNAI2 knockdown RMS cells. **H**, **I** Representative images (**H**) and Graphic representation (**I**) of the migration capacities in the SNAI2 knockdown RMS cells by cell scratching assay. **J**-**M** RD cells with stable knockdown of SNAI2 were implanted subcutaneously into the axillae of nude mice. After 4 weeks, the growth of the tumor was observed with a small animal optical imaging system (**J**), the fluorescence intensity was measured (**K**), ectopic xenograft tumors were excised and photographed (**L**), and the tumor volumes were shown (**M**). **N**, **O** Representative images (**N**) and Graphic representation (**O**) of the IHC staining for SNAI2 and CD34/PAS in nude mice xenograft tumors. CD34 − PAS + represents vasculogenic mimicry (VM). **P**-**R** The zebrafish model was used to analyze the dissemination and metastasis of RD cells (**P**), quantification of migratory tumor numbers (**Q**) and the relative fluorescence density of tumor cells in zebrafish (**R**). Data are presented as the Mean ± SD. ^*^P < 0.05, ^**^P < 0.01, ^***^P < 0.001 and^****^P < 0.0001

To further investigate the biological functions of SNAI2 in RMS, we infected RD and A204 cells with lentiviral vectors to interfere with SNAI2 expression. The knockdown efficiency was validated through RT-PCR and western blotting (Fig. 2A, B). The CCK-8 assay showed reduced proliferation rates in both cells upon SNAI2 inhibition (Fig. 2C). The depletion of SNAI2 also hindered the formation of colonies in RD and A204 cells (Fig. 2D, E). The inhibition of SNAI2 significantly decreased the migratory and invasive capabilities of RMS cells (Fig. 2F-I).

#### In vivo

To investigate the effect of SNAI2 on the proliferation of RMS in vivo, we producing a CDX model using nude mice. The results showed a significant reduction in tumor volume after SNAI2 downregulation (Fig. 2J-M). CD34/PAS IHC analysis showed a significant disparity in the number of VM between the NC and SNAI2 knockdown groups (Fig. 2N, O). To investigate the impact of SNAI2 on RMS metastasis in vivo, we established tumor xenografts in zebrafish embryos.The results indicated that compared to the shSNAI2 group, the NC group showed a significant rise in metastatic tumor cell count (Fig. 2P-R). These results suggest that SNAI2 can increase the proliferation, migration, and VM of RMS.

### SNAI2 promotes VM formation in RMS

**Fig. 3:**
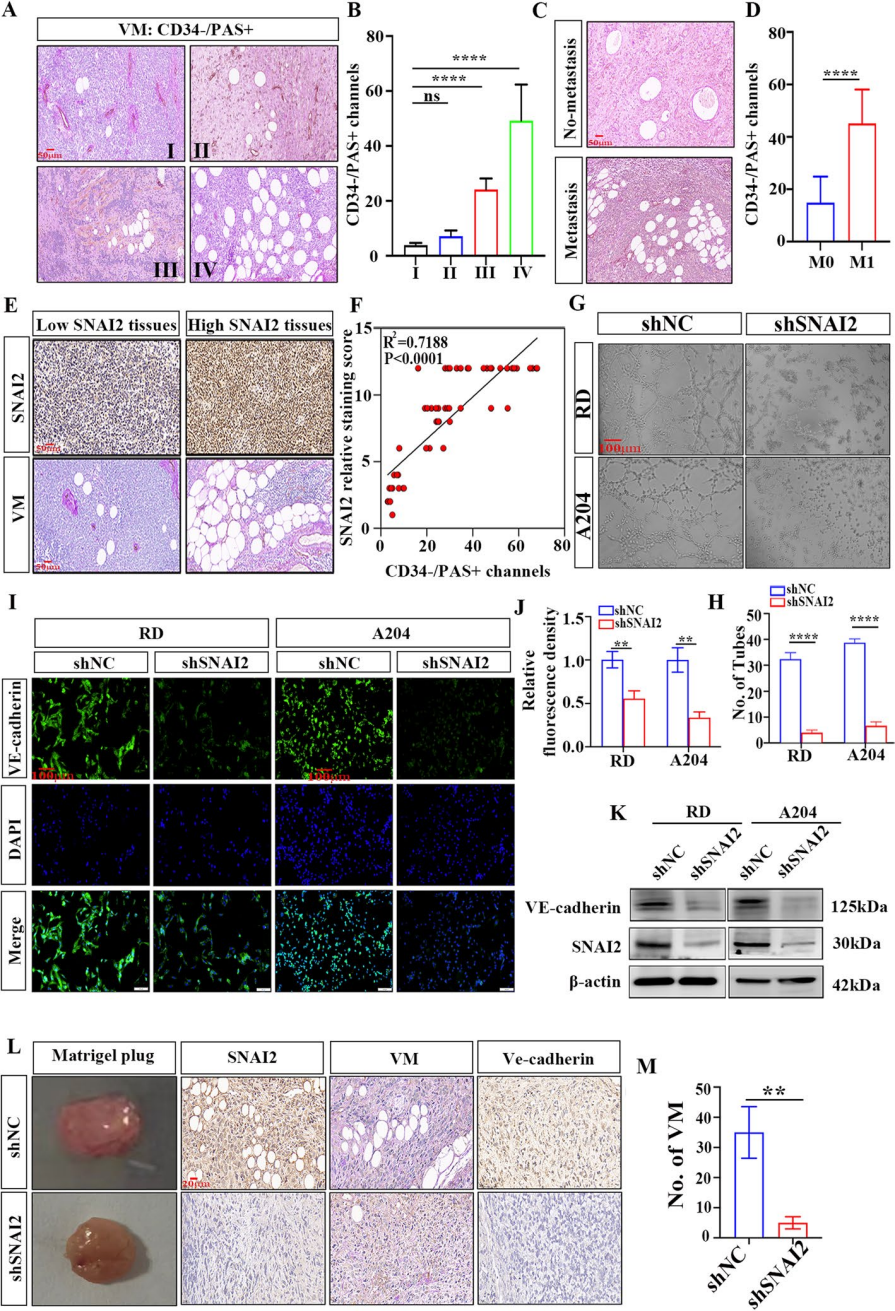
SNAI2 promotes VM formation in RMS cells in vitro and in vivo experiments. **A**, **B** The number of VM associated with tumor stage in RMS patients. **C**, **D** The number of VM in metastasis patients and non-metastasis patients. **E**, **F** Correlation between SNAI2 expression and VM number in human RMS tissues. **G** Tubule formation assay of RD and A204 cells. **H** Column graph in index of tubule formation. **I**, **J** Immunofluorescence revealed that changes in the expression of SNAI2 altered the expression of Ve-cadherin in RMS cells (**I**) and Ve-cadherin protein expression was quantified by the relative mean fluorescence density (**J**). **K** The expression level of Ve-cadherin after knocking down SNAI2 was analyzed by western blot. **L** Representative Matrigel plugs and IHC staining for SNAI2, VM (CD34-/PAS +) tubes, and Ve-cadherin. are shown. **M** Image J software was used to count and analyze tubules. Data are presented as the Mean ± SD. ^**^P < 0.01 and^****^P < 0.0001

This study next investigated the relationship between VM, clinical characteristics, and RMS stage in human samples. Results showed a significant increase in VM quantity in patients in stages III-IV compared to those in stages I-II (Fig. 3A, B) and a greater incidence of VM in patients with metastasis (Fig. 3C, D). The study also examined the link between SNAI2 expression and VM in RMS tissue samples, finding a positive correlation (Fig. 3E, F). In vitro cell tube formation assays showed a decrease in the ability of RMS cells to form tubules (Fig. 3G, H). The results of cell immunofluorescence and western blot experiments suggest that downregulation of SNAI2 expression resulted in decreased expression of VE-cadherin, a molecule associated with VM (Fig. 3I-K). The matrix plug experiment on nude mice showed that the shSNAI2 group had significantly fewer VM than the NC group and a decrease in VE-cadherin expression upon downregulation (Fig. 3L, M). Thus, SNAI2 plays a significant role in promoting VM formation in patients with RMS.

### USP7 deubiquitinates SNAI2

**Fig. 4:**
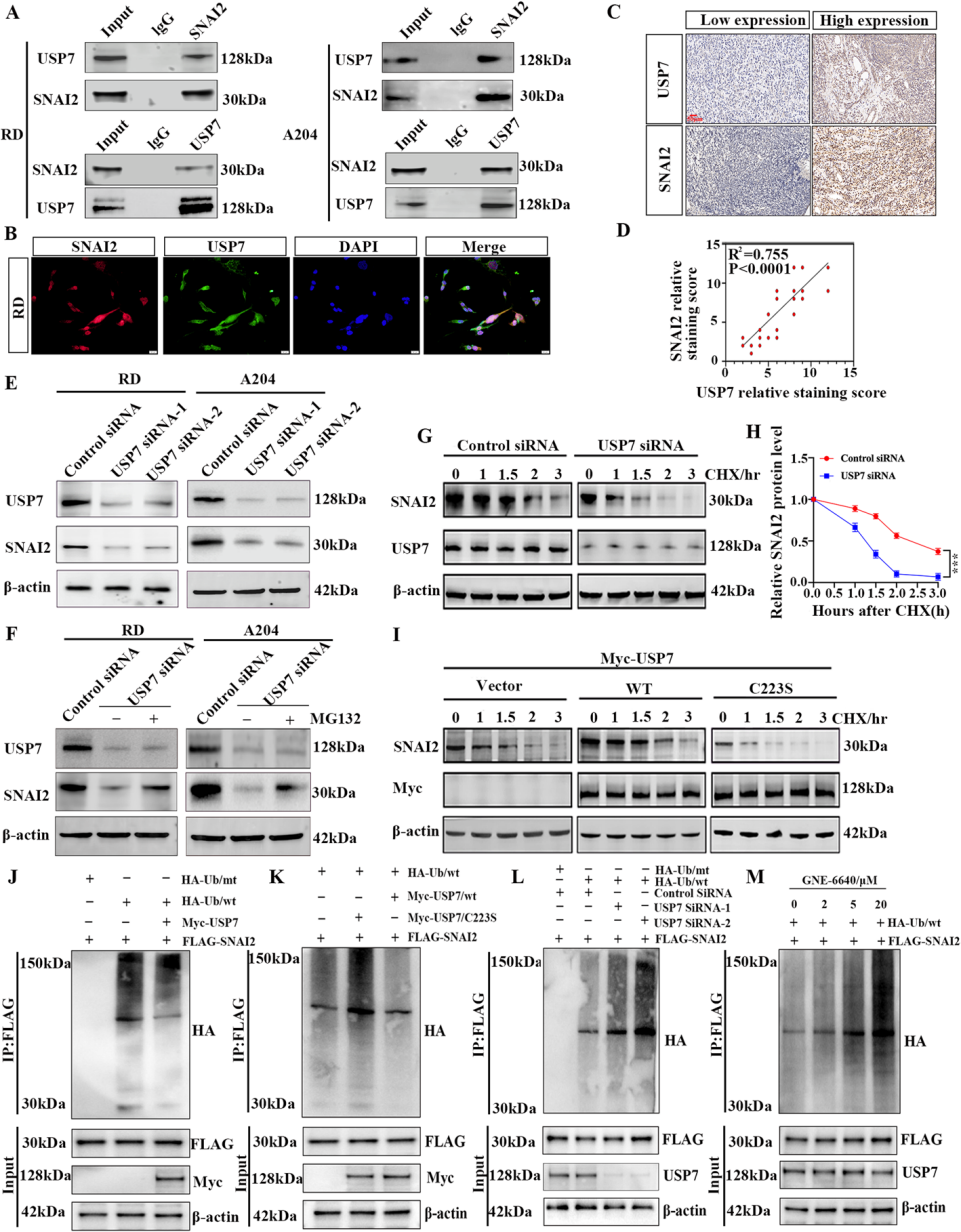
USP7 stabilizes SNAI2 protein levels by deubiquitination. **A** Immunoprecipitation assays showed an interaction between USP7 and SNAI2 Expression in RD and A204 Cells. **B** Immunofluorescence staining of USP7 and SNAI2 in RMS cells was observed using confocal microscopy. **C**, **D** Correlation between USP7 and SNAI2 expression in human RMS tissues. E Effect of USP7 knockdown on the expression of SNAI2 in RD and A204 cells as detected by western blotting. F SNAI2 in USP7 down-regulated RMS cells treated with MG132 (10 μM). G, H SNAI2 protein level in RD cells by downregulating USP7 at the indicated times after CHX (50 µg/mL) addition (**G**) and the plot of relative protein quantification of SNAI2 (**H**). **I** RD cells were transfected with empty Vector of Myc-USP7, WT, or C223S mutants, treated with 50 µg/mL cycloheximide (CHX) at fixed time points, and SNAI2 protein levels were examined by western blot **J**. SNAI2 ubiquitination in Myc-USP7 overexpression RD cells co-transfected with FLAG-SNAI2 and HA-Ub/wt. Cellular extracts were prepared for coimmunoprecipitation assays with anti-FLAG, followed by IB with anti-HA. **K** Effect of Myc-USP7/WT and Myc-USP7/C223S overexpression on SNAI2 ubiquitination in RD cells. **L** RD cells stably expressing FLAG-SNAI2 were cotransfected with control siRNA or USP7 siRNAs together with HA-Ub/wt or HA-Ub/mt as indicated to detect SNAI2 ubiquitination. **M** RD cells stably expressing FLAG-SNAI2 were transfected into HA-Ub/wt cells and cultured in different concentrations of GNE-6640 to detect SNAI2 ubiquitination. Data are presented as the Mean ± SD.^***^P < 0.001

To better understand the biological functions of SNAI2, we utilized affinity purification and mass spectrometry to identify proteins interacting with SNAI2. Due to the lack of known catalytic activity, pharmacologically inhibiting SNAI2 is exceptionally challenging. Therefore, we are considering the search for deubiquitinases that regulate SNAI2 function as an alternative strategy for targeting SNAI2. Combining mass spectrometry results, we identified the deubiquitinase USP7 that binds to SNAI2 (Fig. S2A, B). To further validate the mass spectrometry results, we conducted Co-IP assays to detect the interaction between USP7 and SNAI2. A direct interaction between USP7 and SNAI2 was observed in RMS cells (Fig. 4A). Cellular immunofluorescence analysis showed that SNAI2 and USP7 were colocalized in the nucleus of RMS (Fig. 4B). The results above indicate that SNAI2 can physically interact with USP7.

Subsequently, we examined the expression of USP7 in RMS cells using RT-PCR and western blotting. Results showed that USP7 expression was higher in RD and A204 cells compared to normal HSkMC cells (Fig. S2C and D). Furthermore, we found that USP7 was more abundant in RMS tissues than normal human muscle tissues (Fig. S2E and F). Moreover, USP7 expression was higher in the human RMS metastatic group (Fig. S2G and H). IHC staining and Pearson's correlation analysis also showed a positive correlation between USP7 and SNAI2 expression levels in human RMS tissues (Fig. 4C, D).

To comprehend the mechanism of interaction between SNAI2 and USP7, we investigated the effect of USP7 on SNAI2 expression. Western blot analysis showed a decline in SNAI2 protein levels upon inhibiting USP7 expression using specific siRNAs in RMS cells (Fig. 4E). Conversely, the knockdown of USP7 did not affect SNAI2 mRNA expression (Fig. S2I and J). This suggests that USP7 regulates SNAI2 at the post-translational level without altering its transcription levels. Subsequent results revealed that, following the proteasome-specific inhibitor MG132 treatment, cells with USP7 knockdown exhibited elevated protein levels of SNAI2 compared to the control group (Fig. 4F). These findings suggest that the decrease in USP7 leads to reduced levels of SNAI2 protein, possibly attributed to proteasome-mediated degradation. We next utilized cycloheximide (CHX) chase experiments to assess the ability of USP7 to regulate the stability of SNAI2. Western blot analysis revealed a significant correlation between the decrease in USP7 and the shortened half-life of SNAI2 (Fig. 4G, H, and Fig. S3A). Overexpression of wild-type USP7 prolonged the half-life of SNAI2, whereas overexpression of the catalytically inactive mutant USP7-C223S did not result in an extension of the half-life (Fig. 4I and Fig. S3B, C). These results suggest that the deubiquitination activity of USP7 is essential for increasing SNAI2 stability.

The study investigated if USP7 deubiquitinates SNAI2 in cellular settings. Results showed that upregulation of USP7 reduced SNAI2 ubiquitination levels (Fig. 4J). Experiments confirmed that USP7/C223S mutants could not decrease SNAI2 ubiquitination like USP7/wt (Fig. 4K). The omission of USP7 elevated SNAI2 ubiquitination levels (Fig. 4L). The USP7 inhibitor GNE-6640 showed similar changes in SNAI2 ubiquitination levels to those after USP7 knockout (Fig. 4M). The above results confirm that USP7 can interact with SNAI2 in RMS and mediate deubiquitination to facilitate SNAI2 degradation.

### USP7 knockdown inhibits proliferation, migration, and VM through downregulating SNAI2 in RMS

**Fig. 5:**
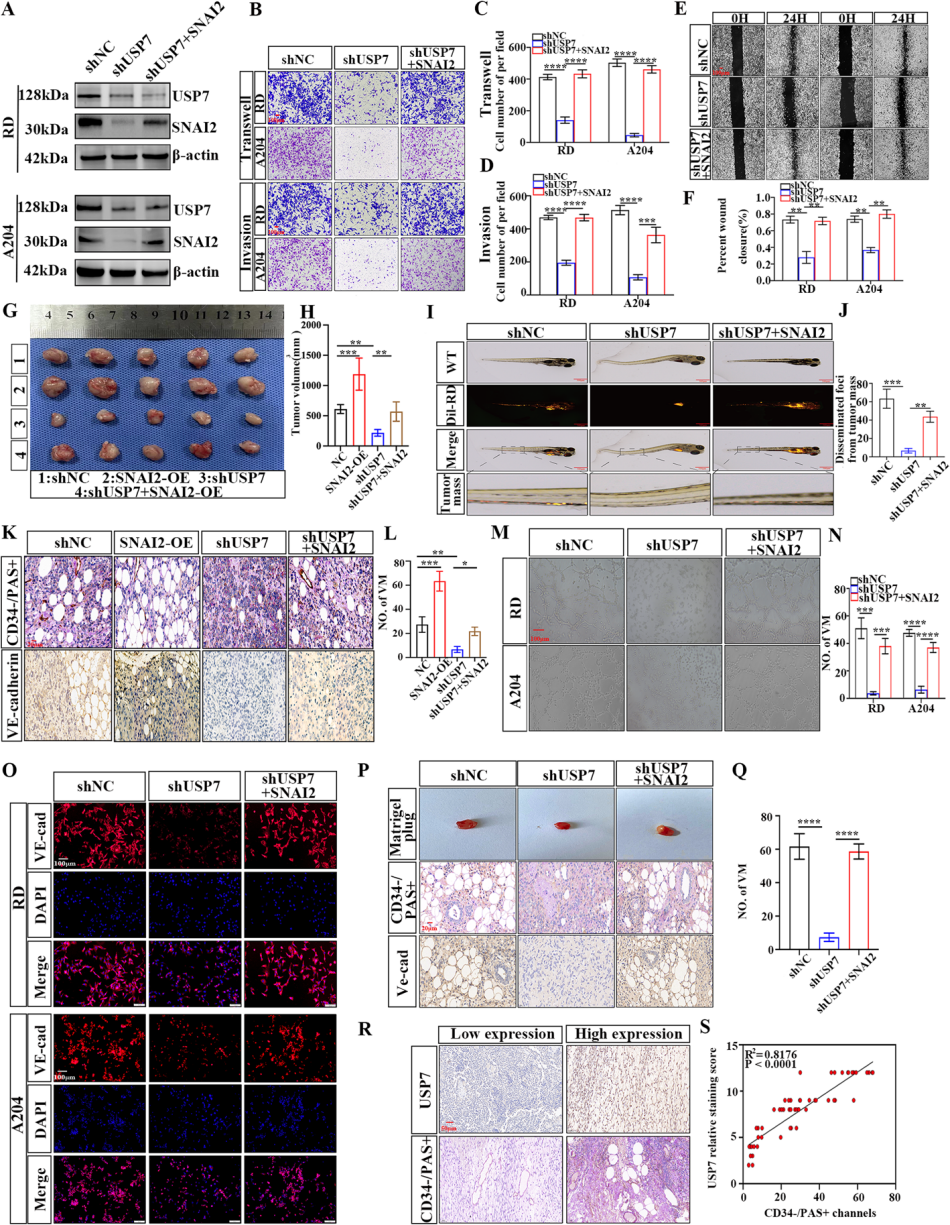
USP7 promotes the proliferation, metastasis, and VM of RMS by up-regulating SNAI2. **A** Western blot analysis verified the expression levels of USP7 and SNAI2 in RMS cells of NC, shUSP7, and shUSP7 + SNAI2 groups. **B** Transwell migration and invasion assay were used to compare the migration and invasion ability of three groups of RMS cells. **C**, **D** Graphic representation of the migration and invasion capacities in the three groups. **E**, **F** Representative images (**E**) and Graphic representation (**F**) of the migration capacities in the three groups of RMS cells by cell scratching assay. **G**, **H** The proliferation ability of the four groups of cells (NC, SNAI2-OE, shUSP7 and shUSP7 + SNAI2 groups) in vivo was verified by subcutaneous tumor formation experiment in nude mice (**G**), and the tumor volume was statistically analyzed (**H**). **I**, **J** The zebrafish model was used to analyze the dissemination and metastasis of the three groups of RMS cells (**I**), and the tumor mass was statistically analyzed (**J**). **K**, **L** Representative images (**K**) and Graphic representation (**L**) of the IHC staining for CD34/PAS and Ve-cadherin of four groups of xenografted tumors in nude mice. **M**, **N** Tubule formation assay of the three groups of RMS cells (**M**), and column graph in index of tubule formation (**N**). **O** Immunofluorescence (IF) revealed changes in the expression of Ve-cadherin in the three groups of RMS cells. **P**, **Q** The ability of VM generation in three groups of RD cells was verified by matrix plug experiment in nude mice (**P**), and the number of tubes was statistically analyzed (**Q**). **R**, **S** IHC analysis of human RMS tissues was conducted to observe the expression of USP7 and VM (**R**), and the correlation analysis of Peason was applied to analyze their correlation (**S**). Data are presented as the Mean ± SD. ^*^P < 0.05, ^**^P < 0.01, ^***^P < 0.001 and^****^P < 0.0001

To validate whether USP7 participates in regulating the malignant biological functions of SNAI2 in RMS, we divided RMS cells into three groups: shNC, shUSP7 and shUSP7 + SNAI2. Western blot analysis confirmed that transfection with the shUSP7 plasmid reduced SNAI2 protein levels in RMS cell lines, while co-transfection of shUSP7 and SNAI2 resulted in an elevation of SNAI2 protein levels in RMS cells (Fig. 5A). CCK-8 and colony formation assays were conducted to validate the proliferative capacity of the three groups of RMS cells. Knockdown of USP7 resulted in a weakened proliferative capacity of RMS cells. However, overexpression of SNAI2 reversed the inhibitory effect of shUSP7 on the proliferative capacity of RMS cells (Fig. S3D-G). To assess the metastasis potential of these three groups of RMS cells, various studies were performed, showing that decreased USP7 expression in RD and A204 cells significantly inhibited RMS cell invasion and metastasis. However, this effect was counteracted by the overexpression of SNAI2 (Fig. 5B-F).

We conducted in vivo experiments to assess the impact of the three groups of cells on proliferation and metastasis. The results indicated that overexpression of SNAI2 significantly promoted tumor growth, while knockdown of USP7 markedly inhibited tumor growth. Moreover, overexpression of SNAI2 rescued the growth suppression mediated by USP7 deficiency in nude mice (Fig. 5G-H). This was also supported in the zebrafish metastasis model, where depleting USP7 reduced the zebrafish's metastatic potential. Overexpression of SNAI2 mitigated the decline in metastatic ability caused by USP7 deficiency in RD cells (Fig. 5I-J). The results suggest that USP7 promotes RMS cell growth and spread through increased SNAI2 expression.

The potential link between USP7 and VM in RMS was also investigated. IHC results of nude mouse tumors showed a decrease in VM formation and VE-cadherin expression in the shUSP7 group compared to the shNC group (Fig. 5K, L). However, the rescue group showed a significant increase in VM and VE-cadherin expression. This led to speculation on USP7's potential to enhance VM in RMS through SNAI2 upregulation. An experiment was conducted to test this potential by overexpressing SNAI2 in RMS cells with USP7 knockdown and assessing VM using a tubule formation assay. The rescue experiment showed that overexpression of SNAI2 restored the decline in tube formation caused by USP7 deletion in RMS cells (Fig. 5M, N). Cell immunofluorescence experiments showed that overexpression partially reversed the reduction of VE-cadherin protein levels in shUSP7-induced RMS cells (Fig. 5O and Fig. S3H). The subcutaneous stromal plug model in nude mice confirmed the role of SNAI2 in USP7-mediated tumor VM in vivo (Fig. 5P, Q). IHC staining showed a positive correlation between USP7 protein levels and VM in human RMS tissues (Fig. 5R, S), suggesting that USP7 promotes VM formation via a SNAI2-dependent mechanism.

### TRIM21 promotes the degradation of SNAI2 protein by ubiquitination in RMS

To further explore the detailed regulatory mechanism underlying SNAI2 ubiquitination, we focused on E3 ubiquitin ligases. By intersecting the mass spectrometry results of SNAI2-co-IPed protein complex (Fig. S2B) and USP7-co-IPed protein complex (Fig. S4A), along with ubiquitin–proteasome-related proteins listed in Genecards, we identified 18 ubiquitination-related proteins (Fig. S4B). Subsequently, we analyzed the interaction network of these 18 proteins using the STRING database, which included the E3 ubiquitin ligase TRIM21 (Fig. S4C). Research has shown that various key tumor suppressors or oncogenes can serve as substrates for TRIM21-mediated ubiquitination, playing crucial roles in malignant behaviors like proliferation and metastasis in multiple cancers.

**Fig. 6:**
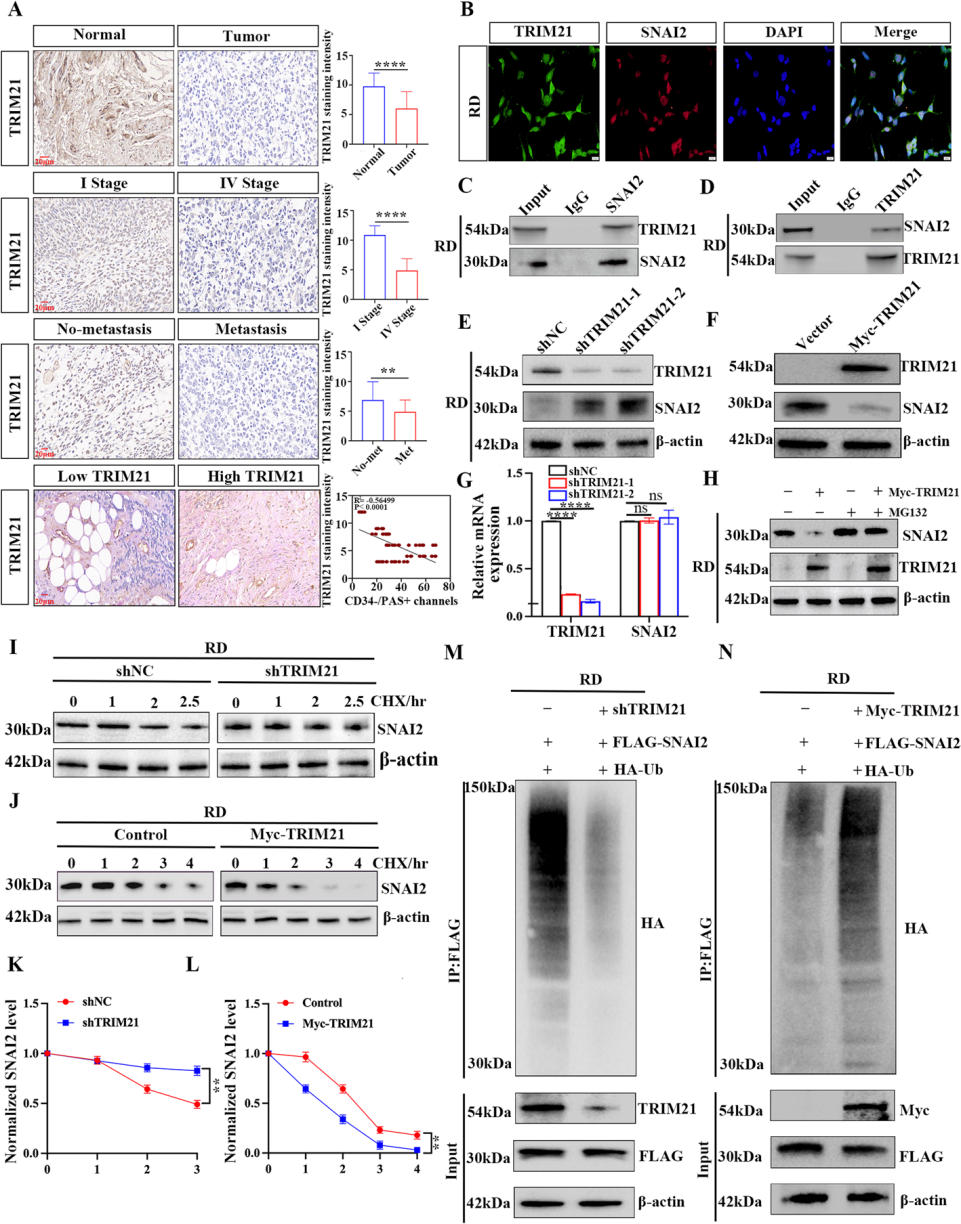
TRIM21 can up-regulate the ubiquitination level of SNAI2 and promote its protein degradation. **A** The expression of TRIM21 in RMS patients and its relationship with clinical features. **B** Immunofluorescence staining assays of USP7 and TRIM21 in RD cells observed by confocal microscopy. **C**, **D** Reciprocal Co-IP assays showed an interaction between USP7 and TRIM21 in RD cells. **E** The effect of downregulation of TRIM21 on SNAI2 expression in RD cells was detected by WB. **F** The effect of overexpression of TRIM21 on SNAI2 protein in RD cells was detected by WB. **G** TRIM21 mRNA expression in RIM21 knockdown and overexpressed RD cells was detected by qRT-PCR. **H** The expression level of SNAI2 in RD cells transfected with TRIM21 overexpression plasmid was detected by WB after co-culture with MG132 (10 μm) for 6 h. **I**, **K** RD cells were transfected with NC or shTRIM21 and treated with 50 µg/mL cycloheximide (CHX) for 1–2.5 h after transfection for 48 h, and then protein was extracted for WB analysis of SNAI2 protein levels (**I**), and the line chart shows quantification of SNAI2 protein levels (**K**). **J**, **L** RD cells were transfected with Vector and Myc-TRIM21 overexpression plasmid, treated with 50 µg/mL cycloheximide (CHX) at a fixed time point, and SNAI2 protein content was detected by WB (**J**), the line chart shows quantification of SNAI2 protein levels (**L**). **M** SNAI2 ubiquitination in TRIM21 knockdown RD cells co-transfected with FLAG-SNAI2 and HA-Ub. N SNAI2 ubiquitination in Myc-TRIM21 overexpression RD cells co-transfected with FLAG-SNAI2 and HA-Ub. Data are presented as the Mean ± SD. ^**^P < 0.01 and^****^P < 0.0001

The study next investigated the relationship between TRIM21 expression and the clinical features of rhabdomyosarcoma (RMS). Results showed reduced TRIM21 expression in RMS cell lines compared to human skeletal muscle cell lines (HSkMCs) (Fig. S4D, E). Immunohistochemistry analysis revealed a significant decrease in TRIM21 expression in tissue samples from patients compared to healthy individuals. Stage IV patients had lower TRIM21 expression than stage I patients. Patients with metastasis showed lower levels of TRIM21 expression. Patients with high TRIM21 scores had a lower density of vasculogenic mimicry (Fig. 6A).

Further research is needed to understand TRIM21's role in RMS pathogenesis and its ability to regulate malignant biological processes by influencing SNAI2 expression. The co-expression of TRIM21 and SNAI2 was confirmed in RMS cell lines via immunofluorescence (Fig. 6B) and CO-IP experiments (Fig. 6C, D). TRIM21 knockout in RD cells increased SNAI2 protein expression (Fig. 6E), whereas SNAI2 mRNA expression remained unaffected (Fig. 6G). The introduction of the Myc-TRIM21 overexpression vector reduced SNAI2 protein expression in RD cells (Fig. 6F). These results suggested that TRIM21 regulates SNAI2 post-translationally without affecting its transcription. The study hypothesized that TRIM21’s regulatory influence on SNAI2 is mediated by regulating its degradation. Overexpressing Myc-TRIM21 in RD cells was treated with MG132, which reversed the TRIM21-induced decrease in SNAI2 protein levels (Fig. 6H). Western blot analysis showed that suppressing TRIM21 in RD cells extended the half-life of SNAI2 (Fig. 6I, K and Fig. S4F), while overexpressing TRIM21 reduced its half-life and protein stability (Fig. 6J, L and Fig. S4G). These results suggest that TRIM21 promotes SNAI2 protein instability through the proteasomal pathway, leading to its degradation. We also investigated TRIM21's role in regulating SNAI2 ubiquitination. Results showed that downregulation of TRIM21 inhibited SNAI2 ubiquitination in RD cells co-transfected with Flag-SNAI2 and HA-Ub plasmids (Fig. 6M), whereas overexpression increased SNAI2 ubiquitination (Fig. 6N). These findings support the hypothesis that TRIM21 regulates ubiquitination levels to enable SNAI2 protein degradation in RMS.

### TRIM21 inhibits VM, proliferation and migration in RMS

**Fig. 7:**
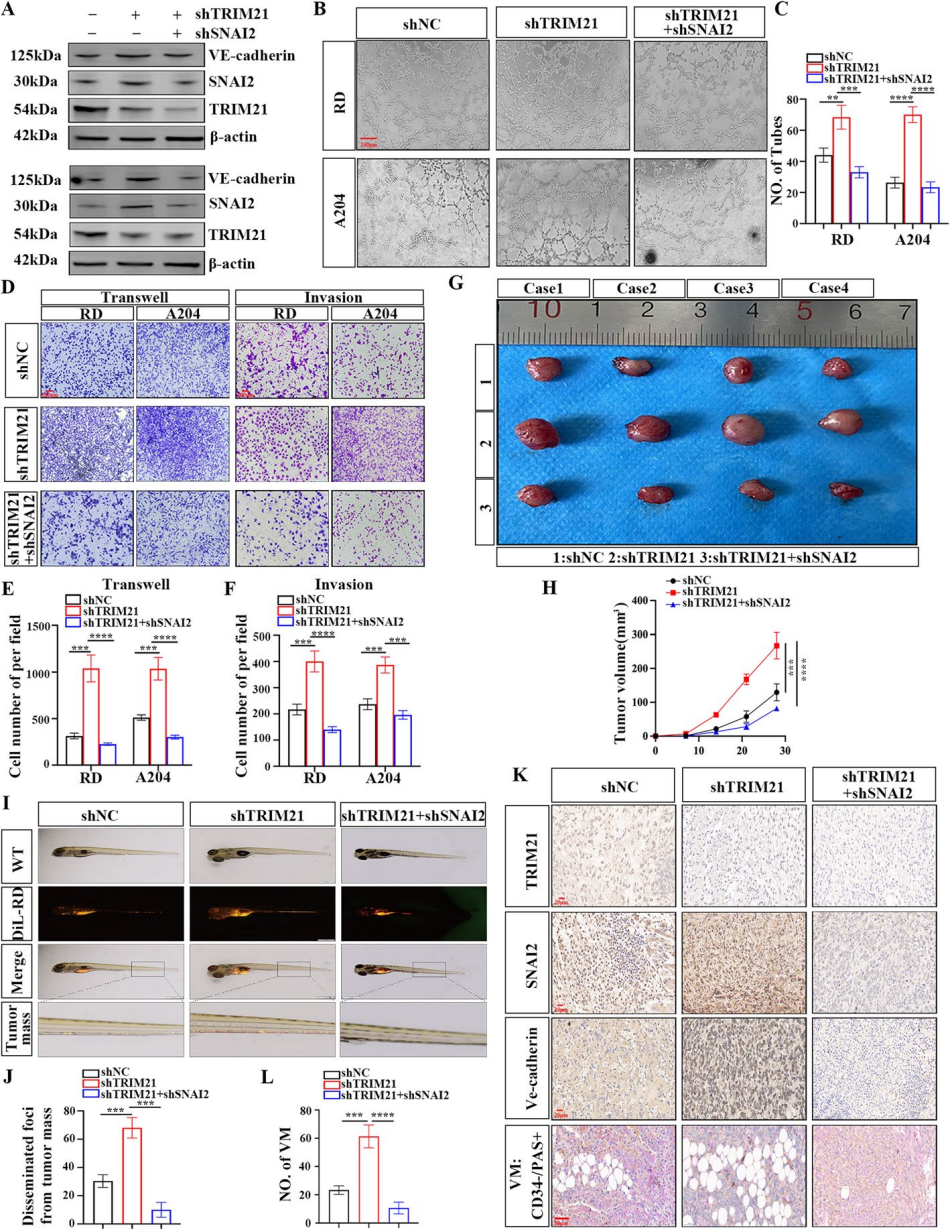
TRIM21 can inhibit VM formation, proliferation, and migration of RMS by regulating SNAI2. **A** WB analysis verified the protein expression levels of TRIM21 and SNAI2 in RMS cells (RD, A204) of NC, shTRIM21, and shTRIM21 + shSNAI2 groups. **B**, **C** Tubule formation assay of the three groups of RMS cells (**B**), and column graph in index of tubule formation (**C**). **D** Transwell migration and invasion assay were used to compare the migration and invasion ability of three groups of RMS cells. **E**, **F** Graphic representation of the migration and invasion capacities in the three groups. **G**, **H** The proliferation abilities of the three groups of cells (NC, shTRIM21, and shTRIM21 + shSNAI2 groups) in vivo was verified by subcutaneous tumor formation experiment in nude mice (**G**), and the tumor volume was statistically analyzed (**H**). **I**, **J** The zebrafish model was used to analyze the dissemination and metastasis of the three groups of RD cells (NC, shTRIM21, shTRIM21 + shSNAI2 groups) (**I**), and the tumor mass was statistically analyzed (**J**). **K**, **L** Representative images (**K**) and Graphic representation (**L**) of the IHC staining for CD34/PAS and Ve-cadherin in three groups of xenografted tumors in nude mice. Data are presented as the Mean ± SD. ^**^P < 0.01, ^***^P < 0.001 and^****^P < 0.0001

Next, this research investigates whether TRIM21 regulates the malignant function of SNAI2 in RMS cells. RMS cells were divided into three groups: NC, shTRIM21, and shTRIM21 + shSNAI2. Western blot analysis confirmed an increase in protein levels of SNAI2 and VE-cadherin in RMS cell lines after transfection with the shTRIM21 plasmid. Co-transfection of shTRIM21 and shSNAI2 reduced SNAI2 and VE-cadherin protein levels in RMS cells (Fig. 7A). The tubuloforming abilities of RD and A204 cells in all three groups showed an increase in tubuloforming capacity following TRIM21 knockdown. However, the impact of shTRIM21 on RMS tubule formation was reversed by SNAI2 knockdown (Fig. 7B, C). The data suggests that TRIM21's boosted cell proliferation could be neutralized by silencing SNAI2 (Fig. S5A-D). Transwell assays showed that TRIM21 produced inhibitory impacts on the migration and invasion of RMS cells by regulating SNAI2 protein expression (Fig. 7D-F).

The CDX model found that the shTRIM21 group had a higher tumor growth rate and volume in nude mice compared to the NC + NC group (Fig. 7G, H). However, the shSNAI2 + shTRIM21 group had a lower tumor growth rate and volume. The zebrafish xenotransplantation model showed that TRIM21 knockdown promoted RD cells' entrance into zebrafish, whereas SNAI2 knockdown reversed this transfer function (Fig. 7I, J). The presence of TRIM21, SNAI2, and VE-cadherin was confirmed in nude mouse tumors. After TRIM21 downregulation, a significant increase in SNAI2 protein content and VE-cadherin level was observed (Fig. 7K, L). These findings suggest that TRIM21 potentially effects RMS proliferation, migration, and VM formation by controlling SNAI2 expression.

### USP7 and TRIM21 function in an antagonistic pattern and contribute to the VM, proliferation, and metastasis of RMS

**Fig. 8:**
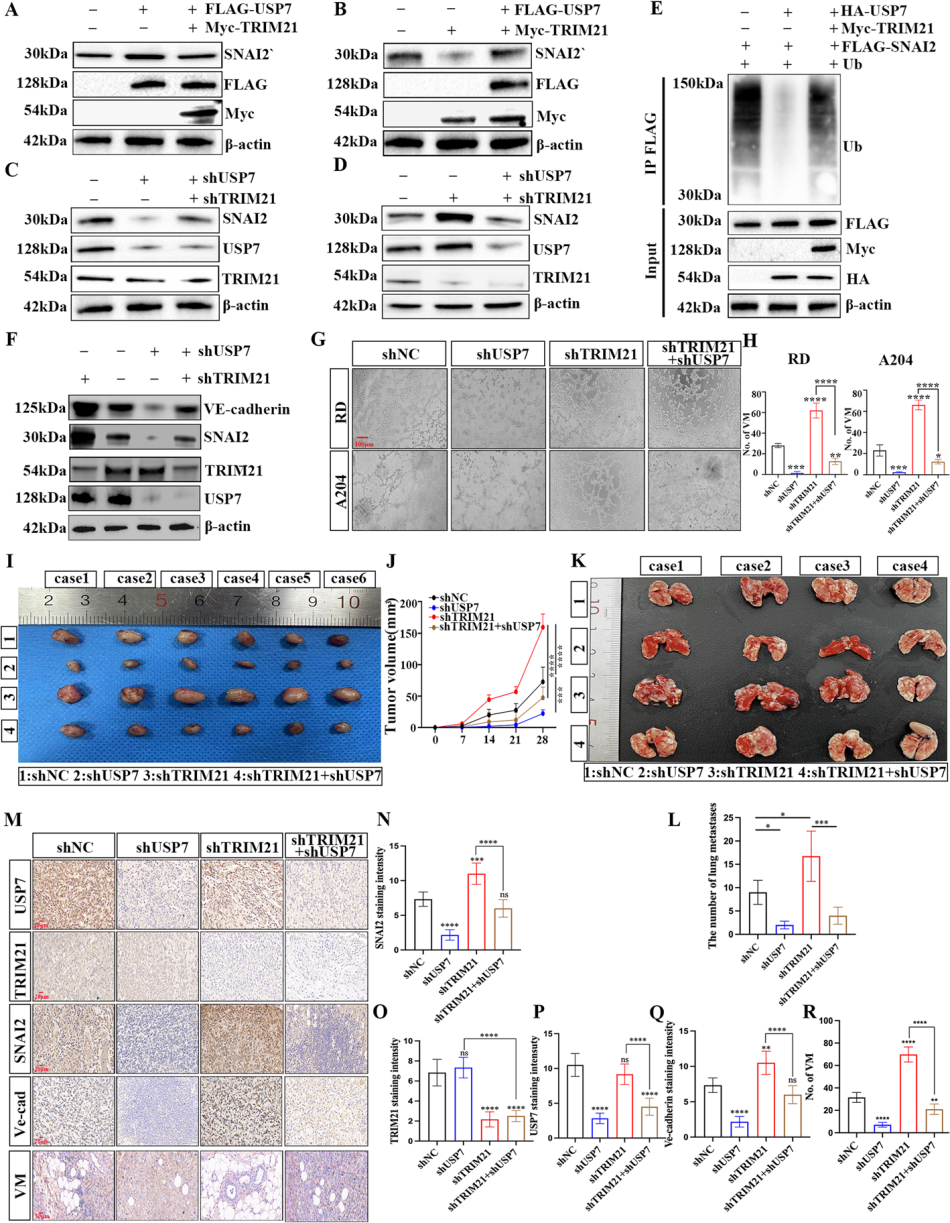
USP7 and TRIM21 regulate VM and progression of RMS by balancing SNAI2 homeostasis. **A**, **B** The protein level of SNAI2 in RD cells co-translated with USP7 and TRIM21 expressing plasmids. **C**, **D** Effect of USP7-knockdown and TRIM21 silencing on the protein level of SNAI2 in RD cells determined by WB. **E** Effect of overexpression of USP7 and TRIM21 on SNAI2 ubiquitination in RD cells. **F** The expression levels of VE-cadherin, SNAI2, TRIM21, and USP7 proteins were verified by WB after co-transfection of shTRIM21 and shUSP7 in RD cells. **G**-**H** Representative images showed the tubule formation assay of the four groups (**G**) and the statistical graph of the number of tubules (**H**) **I**, **J**. The proliferation ability of the four groups of RD cells in vivo was verified by a subcutaneous tumor formation experiment in nude mice (**I**) and the tumor volume was statistically analyzed (**J**). **K**, **L** Representative images showed the lung metastases of different groups (**K**), and the number of nodules in the lung were counted and statistically analyzed (**L**). **M-R** Representative images (**M**) and Graphic representation (**N**-**R**) of the IHC staining for SNAI2, TRIM21, USP7, Ve-cadherin, and CD34-/PAS + of four groups of xenografted tumors in nude mice. Data are presented as the Mean ± SD. ^*^P < 0.05, ^**^P < 0.01, ^***^P < 0.001 and^****^P < 0.0001

Given the pivotal roles of USP7 and TRIM21 in regulating SNAI2 ubiquitination, we hypothesize that USP7 and TRIM21 may collectively modulate SNAI2 expression, potentially impacting the vascular mimicry of RMS. To validate the regulatory relationship between USP7 and TRIM21 on SNAI2 expression, we co-transfected RD cells with overexpression plasmids of USP7 and TRIM21, and then verified the expression efficiency using RT-PCR and Western blot (Fig. S6A, B and Fig. 8A, B). We observed that the mRNA and protein expression levels of USP7 and TRIM21 did not reciprocally influence each other. Overexpressing USP7 led to an increase in SNAI2 protein content, while overexpressing TRIM21 counteracted this effect (Fig. 8A). Overexpressing TRIM21 significantly reduced SNAI2 protein expression, and transfecting with the overexpression USP7 plasmid restored SNAI2 protein content (Fig. 8B). Subsequently, we co-transfected RD cells with shUSP7 and shTRIM21 plasmids, followed by validation of expression efficiency using RT-PCR and Western blot (Fig. S6C, D and Fig. 8C, D). After transfection with shUSP7, the protein content of SNAI2 decreased, whereas the protein expression level of SNAI2 could be reversed after co-transfection with shTRIM21 (Fig. 8C). Furthermore, the protein expression of SNAI2 was significantly up-regulated after TRIM21 was knocked down, and the protein content of SNAI2 was decreased after co-transfection with shUSP7 (Fig. 8D). The findings suggest that USP7 and TRIM21 can maintain the stability of SNAI2 protein level. Moreover, the regulation of SNAI2 ubiquitination by USP7 and TRIM21 in RD cells. Results showed an attenuation in ubiquitination after USP7 overexpression (Fig. 8E), but reversed upon transfection with TRIM21, increasing ubiquitin levels.

Considering that SNAI2 protein is a common target of USP7 and TRIM21, we then attempted to ascertain whether this balancing mechanism is associated with the vasculogenic mimicry and malignant progression of RMS. Western blot analysis revealed that TRIM21 knockdown increased SNAI2 and VE-cadherin expression, whereas USP7 knockdown decreased these expressions. The combined downregulation of TRIM21 and USP7 reversed the expression levels of SNAI2 and VE-cadherin (Fig. 8F). Tubuloplasty experiments showed that RMS cells with shTRIM21 increased tubule number, which was mitigated by USP7 downregulation (Fig. 8G, H). The above experiments confirm USP7 and TRIM21 function in an antagonistic pattern contribute to the VM formation of RMS in vitro.

We further evaluated the roles of USP7 and TRIM21 in the in vivo progression of RMS. Downregulation of USP7 expression significantly suppressed tumor growth in mice, whereas reduced TRIM21 expression promoted xenograft tumor growth. Suppression of TRIM21 antagonized tumor suppression induced by USP7 downregulation. USP7 knockdown hindered the inhibition of tumor proliferation induced by ShTRIM21 (Fig. 8I, J). IHC detection on nude mouse tumors revealed a significant reversal of the decreased expression of SNAI2, VE-cadherin, and VM number induced by USP7 knockdown through the downregulation of TRIM21. Conversely, USP7 knockdown reversed the increase in SNAI2, VE-cadherin expression, and VM number induced by TRIM21 downregulation (Fig. 8M-R).

The study examined the impact of USP7 and TRIM21 on the metastatic potential of RMS cells in vivo. Results showed that reducing USP7 expression decreased the metastasis of tumor cells in Zebrafish, while downregulating TRIM21 increased tumor metastasis and reversed the inhibition of metastasis caused by shUSP7 cells. Downregulating USP7 also suppressed the transfer induced by shTRIM21 (Fig. S6E, F). The study also established a lung metastasis model in nude mice, confirming the outcomes were consistent with those observed in the zebrafish model (Fig. 8K, L). These findings contribute to a better understanding of the novel mechanisms by which USP7 and TRIM21, in an antagonistic manner, regulate SNAI2 stability, thereby impacting the angiogenic mimicry and malignant progression of RMS.

## Discussion

The current treatment strategies for metastatic and recurrent RMS are insufficient, and understanding the molecular mechanisms behind RMS progression is crucial [1]. High levels of SNAI2 have been found to accelerate tumor progression [51, 52], but its function in VM formation remains unclear. This study presents clinical, in vitro, and animal evidence supporting SNAI2 as a key driver of VM formation in RMS.

Although SNAI2 plays a key regulatory role in RMS, there is currently no effective pharmacological pathway to directly target SNAI2. Given the easier synthesis of deubiquitinating enzymes (DUBs), recognizing and stabilizing SNAI2 DUBs could serve as a strategy to develop new drug targets to reduce SNAI2 levels and inhibit the progression of RMS. While there is extensive literature on the regulation of SNAI2 protein ubiquitination, including the ubiquitin ligases such as FBXO28 [53] and deubiquitinases such as USP20 [39], effective targets for SNAI2 regulation remain elusive, particularly regarding its mechanisms in RMS. We detected the SNAI2-Co-IPed protein complex by mass spectrometry and screened out the deubiquitinating enzyme USP7. we then conducted CO-IP and IF experiments to confirm the interaction between USP7 and SNAI2 in RD and A204 cells. Subsequently, our results showed a significant reduction in SNAI2 protein levels upon USP7 knockout, whereas mRNA levels remained relatively unchanged. This suggests that the decrease in SNAI2 levels due to USP7 depletion is likely due to proteasome-mediated protein degradation mechanisms. The relationship between USP7 and SNAI2 stability, investigated through half-life experiments, showed that USP7's deubiquitination activity is crucial for stabilizing SNAI2. The study also evaluated USP7's expression level in RMS patients and its correlation with pathological characteristics. A significant increase in USP7 expression was associated with unfavorable pathological characteristics. In vitro and in vivo functional rescue experiments showed that overexpression of SNAI2 reversed the inhibitory effects on proliferation and metastasis caused by USP7 depletion. USP7 also facilitates VM in RMS in a SNAI2-dependent manner.

Ubiquitination and deubiquitination are two distinct post-translational modification processes that involve the attachment and detachment of ubiquitin from target proteins, respectively. To further explore the detailed regulatory mechanism underlying SNAI2 ubiquitination, we focused on E3 ubiquitin ligases. By intersecting the mass spectrometry results of SNAI2-co-IPed protein complex and USP7-co-IPed protein complex, along with ubiquitin–proteasome-related proteins listed in Genecards, we identified the E3 ubiquitin ligase TRIM21. Studies have shown that TRIM21 inhibits the progression of EMT by regulating the ubiquitination and degradation of Snail, the master regulator of EMT. Its mutation eliminates TRIM21-mediated Snail ubiquitination and degradation, thereby increasing breast cancer cell migration and invasion [54]. Therefore, we speculate whether the E3 ligase TRIM21, which has the function of ubiquitin, has a regulatory effect on the ubiquitization level of SNAI2 in RMS. We first demonstrated that SNAI2 interacts with TRIM21 in RMS cells. IHC analysis reveals higher TRIM21 expression in RMS tissues compared to normal muscle tissues. The RMS cell line shows reduced TRIM21 mRNA levels compared to the human skeletal muscle cell line. The ubiquitination of SNAI2 by TRIM21 reduces its stability, inhibiting VM and malignant progression of RMS. This study enhances our understanding of TRIM21's regulatory mechanism in the SNAI family and the role of the ubiquitin–proteasome system in tumor development. Clarifying the mechanism of action, focusing on SNAI2 protein expression by TRIM21, offers a potential target for clinical use of anti-VM.

The above studies demonstrated that USP7 and TRIM21 proteases with ubiquitination modification regulation have a common target protein SNAI2. So we further explored their combined regulatory relationship on SNAI2 expression. We confirmed that USP7 and TRIM21 play a key role in controlling SNAI2 stability in an antagonistic rather than competitive manner, thus influencing VM formation in RMS. This study substantially complements the regulation of SNAI2 expression in tumors and helps identify new therapeutic targets for RMS.

## Conclusion

In summary, this study presents a new method for regulating the malignant progression of RMS by balancing SNAI2 ubiquitination and deubiquitination. The deubiquitination enzyme USP7 and the E3 ligase TRIM21 control this pathway, affecting VM formation, proliferation and migration of RMS cells. This new mechanism may offer novel insights and approaches to inhibit the malignant progression of RMS patients with high expression levels of SNAI2 protein. Additionally, the regulation of vasculogenic mimicry through the targeting of SNAI2, USP7, and TRIM21 presents a potential therapeutic strategy for the treatment of RMS. The efficacy of small molecule USP7 inhibitors has been validated in vivo. Combining targeted small molecule inhibitors of VM formation with anti-angiogenic therapy represents a promising therapeutic approach.

### Supplementary Information

**Additional file 1: Supplemental Figure 1.** RMS cells can form VM. A The experiments on cell tubule formation demonstrated that RMS cell lines can generate tubules within the matrix gel, similar to vascular endothelial cells. B The stromal plug test conducted on nude mice revealed that RD and A204 cells could create lumen channels resembling those found in endothelial cells HUVEC. The PAS+ lumen formed by tumor cells showed CD34-, suggesting the existence of VM. Conversely, the PAS+ lumen formed by endothelial cells exhibited CD34+, indicating the presence of blood vessels.

**Additional file 2: Supplemental Figure 2.** Identifying the potential binding protein USP7 interacting with SNAI2 and detecting its expression in RMS. A, B Immunoaffinity purification and mass spectrometry of SNAI2-containing protein complexes. Whole-cell extracts from RD cells expressing stably integrated FLAG-SNAI2 were purified with an anti-FLAG affinity column and visualized by silver staining following SDS-PAGE (A); The protein bands in the gel were recovered and analyzed by mass spectrometry, and the coverage of the indicated proteins is shown (B). C The mRNA expression of USP7 in RMS cells and HSkMC cells was detected by RT-PCR. D The mRNA expression of USP7 in RMS cells and HSkMC cells was detected by WB. E, F. The expression level of USP7 in 59 RMS tissues and 12 normal tissues. G, H The expression level of SNAI2 in metastasis tissues and no-metastasis tissues. I, J The effect of down-regulated USP7 on SNAI2 mRNA expression in RD (I) and A204 cells (J) was detected by RT-PCR. Data are presented as the Mean ± SD. ^****^P < 0.0001.

**Additional file 3: Supplemental Figure 3.** USP7 promotes the proliferation of RMS cells by up-regulating SNAI2 in vitro. A The statistical bar chart of half-life time for Figure 4G. B, C The plot of relative protein quantification of SNAI2 (B) and the statistical bar chart of half-life time for Figure 4I (C) D, E CCK8 assay verified the proliferation ability in RD and A204 cells of NC group, shUSP7 group, and shUSP7+SNAI2 group. F, G Colony formation assay verified the proliferation ability of RD and A204 cells of NC group, shUSP7 group, and shUSP7+SNAI2 group (F); The colony counts were normalized to the control and expressed as a percentage, and results are represented in the bar graph (G). H Ve-cadherin protein expression of Figure 5O was quantified by the relative mean fluorescence density. Data are presented as the Mean ± SD. ^*^P< 0.05, ^**^P  < 0.01, ^***^P < 0.001 and ^****^P < 0.0001.

**Additional file 4: Supplemental Figure 4.** Identifying the potential binding protein TRIM21 and detecting its expression in RMS cells. A The MS basepeak of the USP7-co-IPed protein complex. B The protein obtained by intersecting the mass spectrometry results of SNAI2-co-IPed protein complex and USP7-co-IPed protein complex, along with ubiquitin-proteasome-related proteins listed in Genecards. C The relationship diagram of 18 ubiquitination related proteins in the STRING database. D The mRNA expression of TRIM21 in RMS and HSkMC cells was detected by RT-PCR. E The protein expression of TRIM21 in RMS cells and HSkMC cells was detected by WB. F The statistical bar chart of half-life time for Figure 6I. G The statistical bar chart of half-life time for Figure 6J. Data are presented as the Mean ± SD. ^**^P < 0.01 and ^****^P < 0.0001.

**Additional file 5: Supplemental Figure 5.** TRIM21 can inhibit the proliferation of RMS by regulating SNAI2 in vitro. A, B CCK8 assay verified the proliferation ability in RD and A204 cells of NC group, shTRIM21 group, and shTRIM21+shSNAI2 group. C Colony formation assay verified the proliferation ability of RD and A204 cells of NC group, shTRIM21 group, and shTRIM21+shSNAI2 group. D The colony counts were normalized to the control and expressed as a percentage, and results are represented in the bar graph.Data are presented as the Mean ± SD. ^**^P  < 0.01, ^***^P < 0.001 and ^****^P < 0.0001.

**Additional file 6: Supplemental Figure 6.** TRIM21 can inhibit proliferation of RMS by regulating SNAI2 in vitro. A, B RT-PCR indicated that the mRNA expression levels of USP7 and TRIM21 in RD cells co-translated with USP7 and TRIM21 expressing plasmids. C, D The mRNA levels of USP7 and TRIM21 were analyzed by RT-PCR in RD Cells. E, F The zebrafish model was used to analyze the dissemination and metastasis of the four groups of RD cells (E) and the tumor mass was statistically analyzed (F).Data are presented as the Mean ± SD. ^****^P < 0.0001.

## Data Availability

All data generated or analyzed during this study are included in the published article.

## Abbreviations

RMS: Rhabdomyosarcoma
VM: Vasculogenic mimicry
USP7: Ubiquitin-specific protease 7
CDX: Cell-derived xenograft
EMT: Epithelial-mesenchymal transition
DUB: Deubiquitination enzyme
WB: Western blotting
PAS: Periodic acid-schiff
EMT: Epithelial-mesenchymal transition
IF: Immunofluorescent
IHC: Immunohistochemistry
HSkMCs: Human skeletal muscle cells
HUVEC: Human vascular endothelial cells
