# Supplementary figures and images for: The deubiquitinase USP7 and E3 ligase TRIM21 regulate vasculogenic mimicry and malignant progression of RMS by balancing SNAI2 homeostasis
Source: J Exp Clin Cancer Res. 2024 May 4;43:135. doi: 10.1186/s13046-024-03056-1 (PMC11069146; doi:10.1186/s13046-024-03056-1)

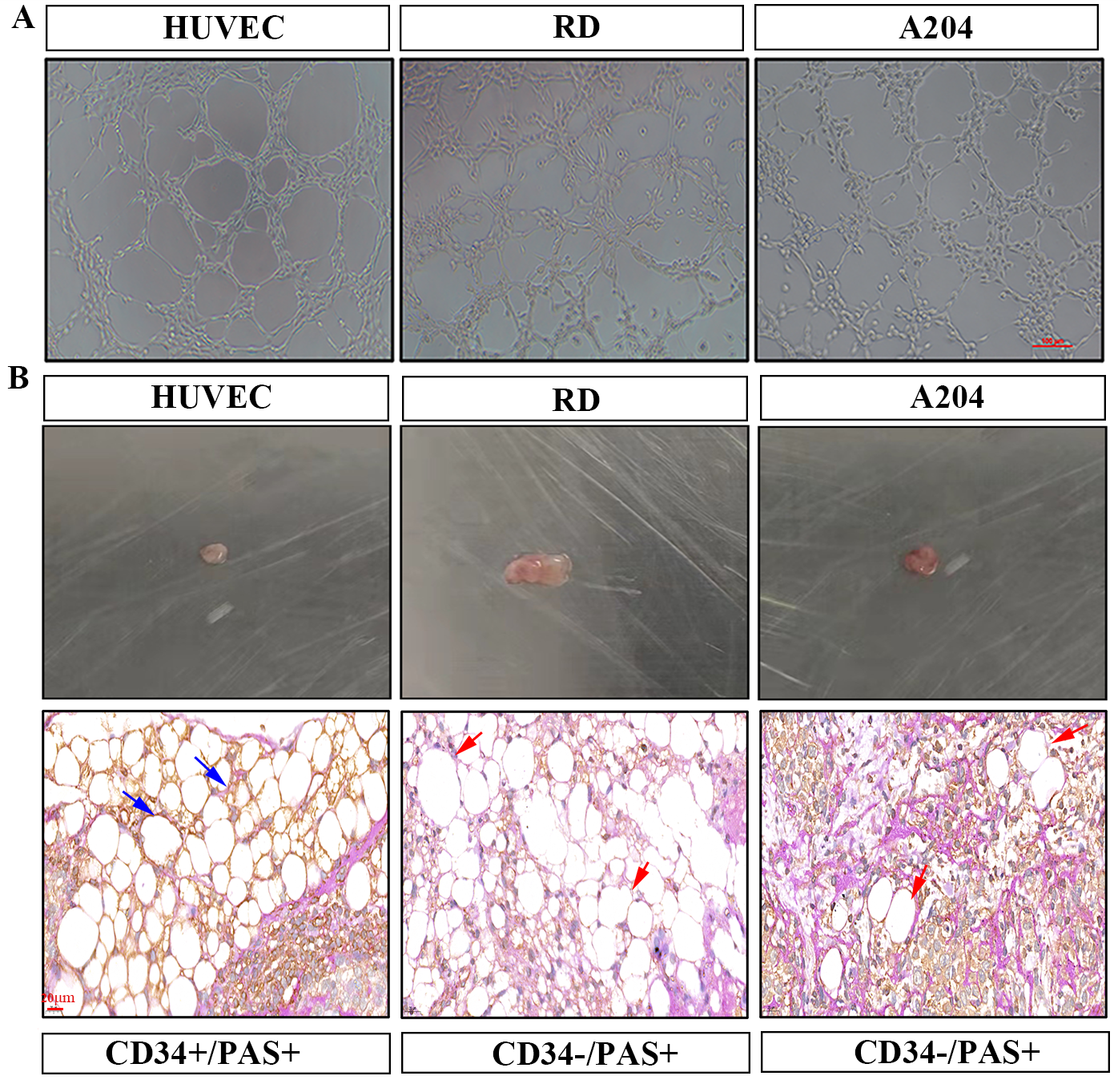

Supplement: Supplementary file 1 — Additional file 1: Supplemental Figure 1. RMS cells can form VM. A The experiments on cell tubule formation demonstrated that RMS cell lines can generate tubules within the matrix gel, similar to vascular endothelial cells. B The stromal plug test conducted on nude mice revealed that RD and A204 cells could create lumen channels resembling those found in endothelial cells HUVEC. The PAS+ lumen formed by tumor cells showed CD34-, suggesting the existence of VM. Conversely, the PAS+ lumen formed by endothelial cells exhibited CD34+, indicating the presence of blood vessels. [file 13046_2024_3056_MOESM1_ESM.png]

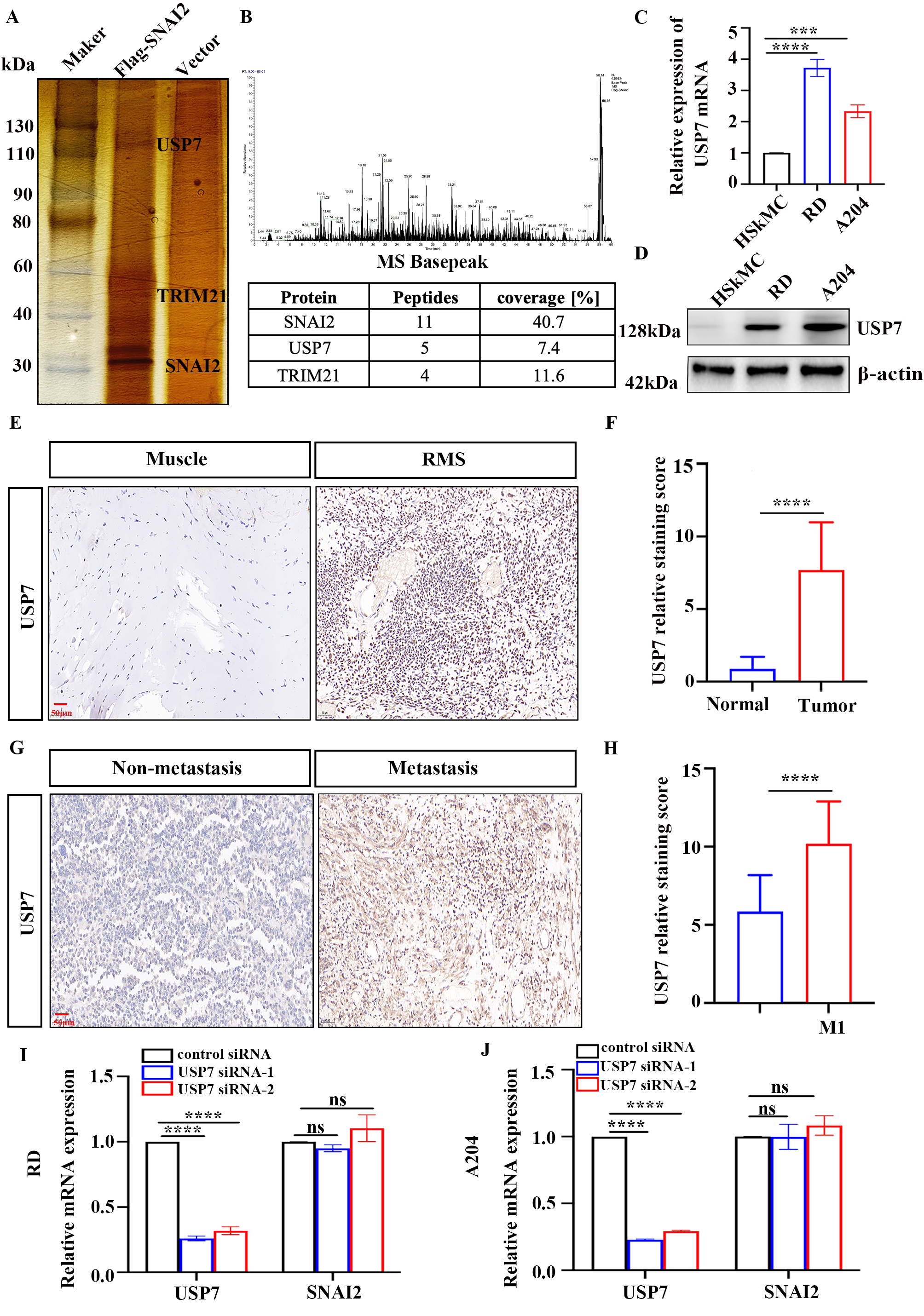

Supplement: Supplementary file 2 — Additional file 2: Supplemental Figure 2. Identifying the potential binding protein USP7 interacting with SNAI2 and detecting its expression in RMS. A, B Immunoaffinity purification and mass spectrometry of SNAI2-containing protein complexes. Whole-cell extracts from RD cells expressing stably integrated FLAG-SNAI2 were purified with an anti-FLAG affinity column and visualized by silver staining following SDS-PAGE (A); The protein bands in the gel were recovered and analyzed by mass spectrometry, and the coverage of the indicated proteins is shown (B). C The mRNA expression of USP7 in RMS cells and HSkMC cells was detected by RT-PCR. D The mRNA expression of USP7 in RMS cells and HSkMC cells was detected by WB. E, F. The expression level of USP7 in 59 RMS tissues and 12 normal tissues. G, H The expression level of SNAI2 in metastasis tissues and no-metastasis tissues. I, J The effect of down-regulated USP7 on SNAI2 mRNA expression in RD (I) and A204 cells (J) was detected by RT-PCR. Data are presented as the Mean ± SD. ****P < 0.0001. [file 13046_2024_3056_MOESM2_ESM.png]

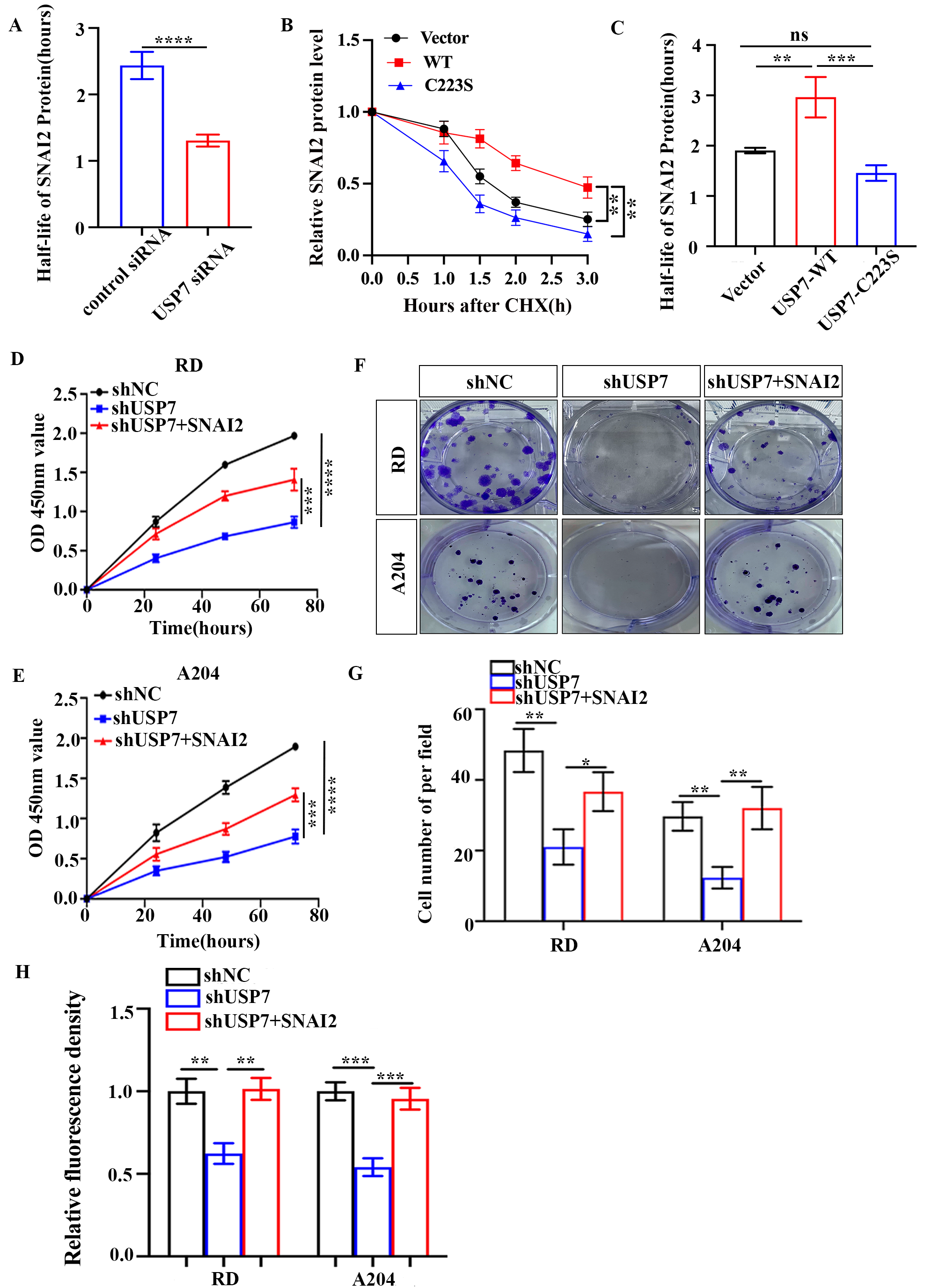

Supplement: Supplementary file 3 — Additional file 3: Supplemental Figure 3. USP7 promotes the proliferation of RMS cells by up-regulating SNAI2 in vitro. A The statistical bar chart of half-life time for Figure 4G. B, C The plot of relative protein quantification of SNAI2 (B) and the statistical bar chart of half-life time for Figure 4I (C) D, E CCK8 assay verified the proliferation ability in RD and A204 cells of NC group, shUSP7 group, and shUSP7+SNAI2 group. F, G Colony formation assay verified the proliferation ability of RD and A204 cells of NC group, shUSP7 group, and shUSP7+SNAI2 group (F); The colony counts were normalized to the control and expressed as a percentage, and results are represented in the bar graph (G). H Ve-cadherin protein expression of Figure 5O was quantified by the relative mean fluorescence density. Data are presented as the Mean ± SD. *P< 0.05, **P < 0.01, ***P < 0.001 and ****P < 0.0001. [file 13046_2024_3056_MOESM3_ESM.png]

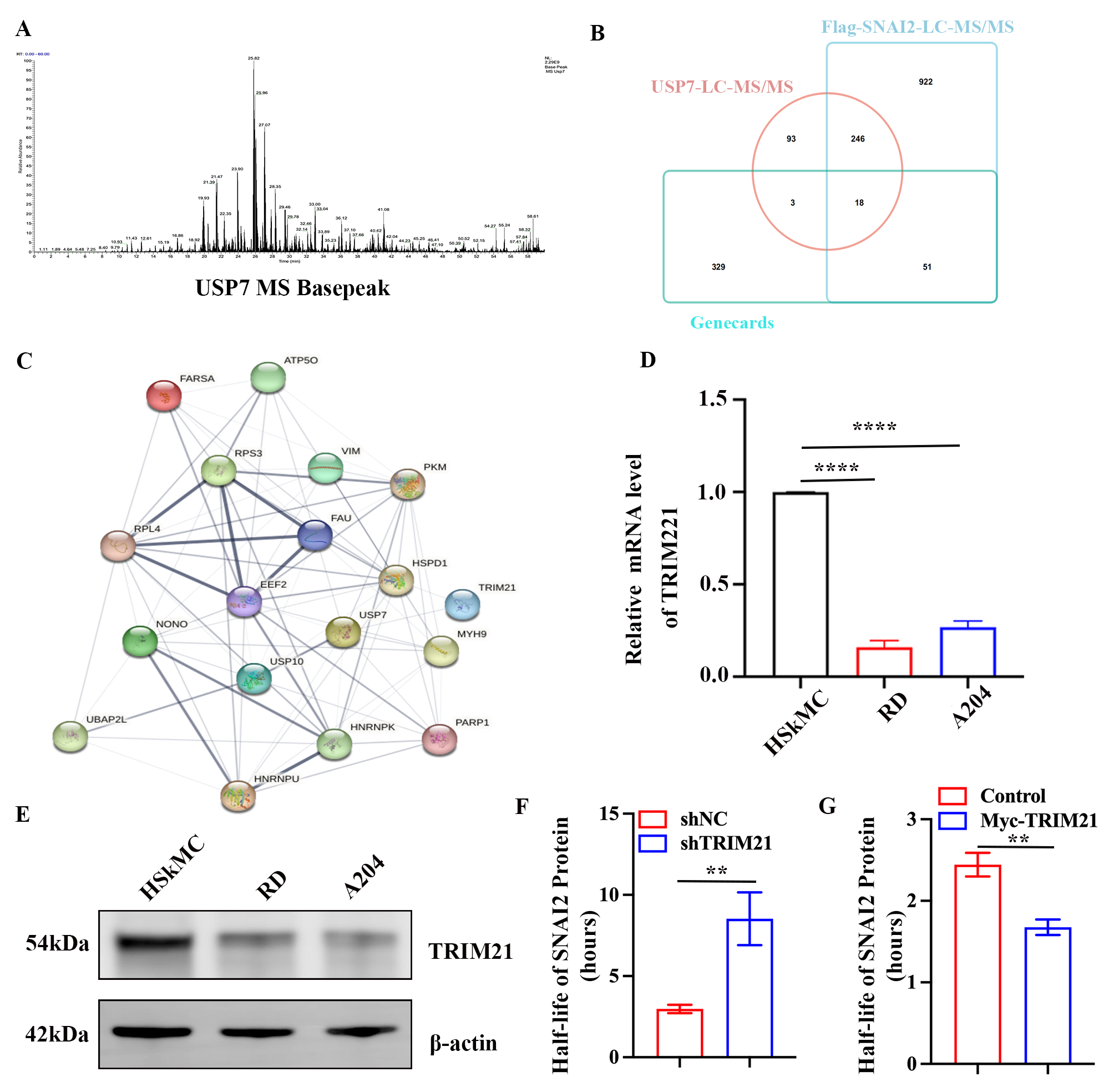

Supplement: Supplementary file 4 — Additional file 4: Supplemental Figure 4. Identifying the potential binding protein TRIM21 and detecting its expression in RMS cells. A The MS basepeak of the USP7-co-IPed protein complex. B The protein obtained by intersecting the mass spectrometry results of SNAI2-co-IPed protein complex and USP7-co-IPed protein complex, along with ubiquitin-proteasome-related proteins listed in Genecards. C The relationship diagram of 18 ubiquitination related proteins in the STRING database. D The mRNA expression of TRIM21 in RMS and HSkMC cells was detected by RT-PCR. E The protein expression of TRIM21 in RMS cells and HSkMC cells was detected by WB. F The statistical bar chart of half-life time for Figure 6I. G The statistical bar chart of half-life time for Figure 6J. Data are presented as the Mean ± SD. **P < 0.01 and ****P < 0.0001. [file 13046_2024_3056_MOESM4_ESM.png]

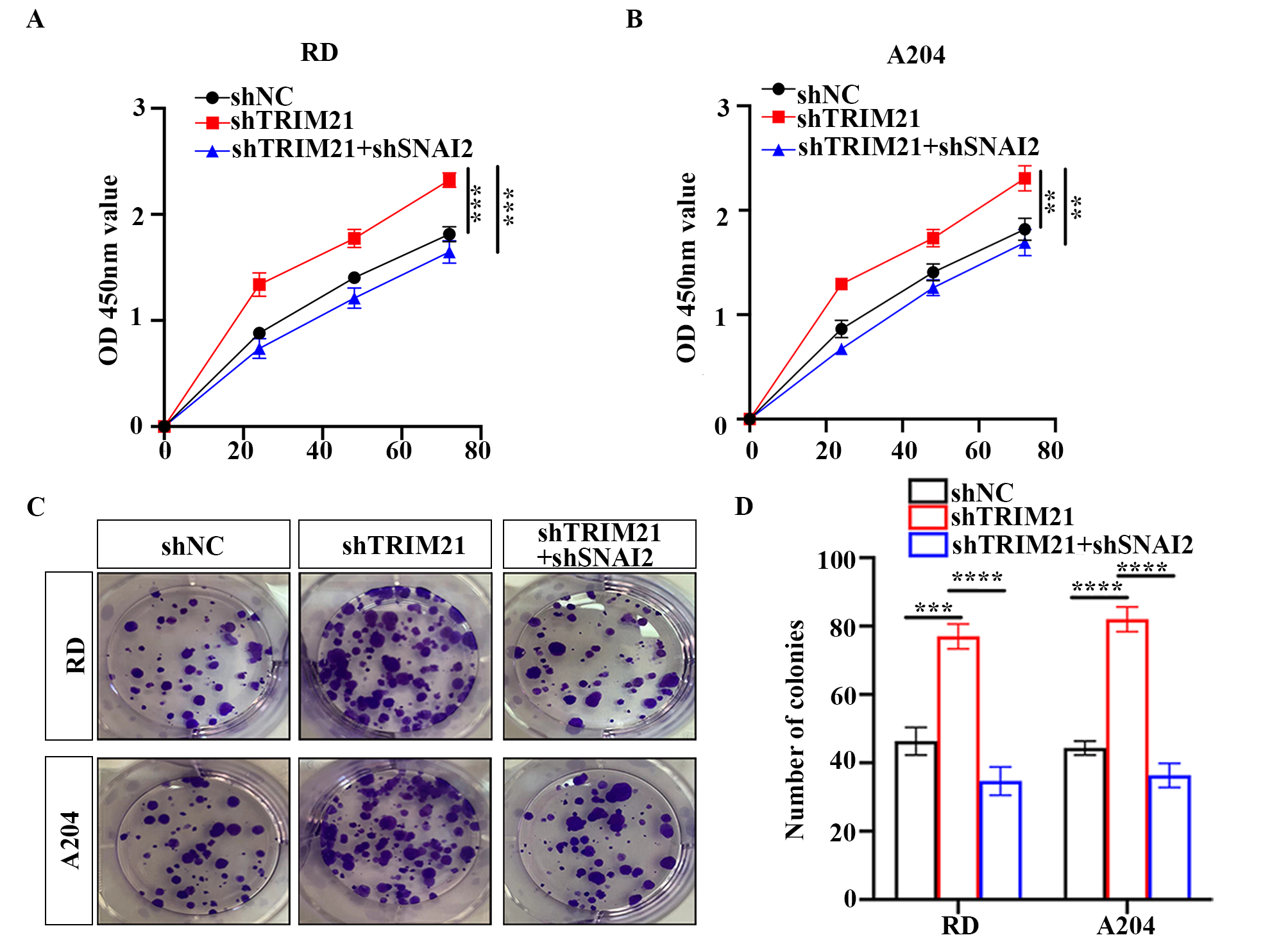

Supplement: Supplementary file 5 — Additional file 5: Supplemental Figure 5. TRIM21 can inhibit the proliferation of RMS by regulating SNAI2 in vitro. A, B CCK8 assay verified the proliferation ability in RD and A204 cells of NC group, shTRIM21 group, and shTRIM21+shSNAI2 group. C Colony formation assay verified the proliferation ability of RD and A204 cells of NC group, shTRIM21 group, and shTRIM21+shSNAI2 group. D The colony counts were normalized to the control and expressed as a percentage, and results are represented in the bar graph.Data are presented as the Mean ± SD. **P < 0.01, ***P < 0.001 and ****P < 0.0001. [file 13046_2024_3056_MOESM5_ESM.png]

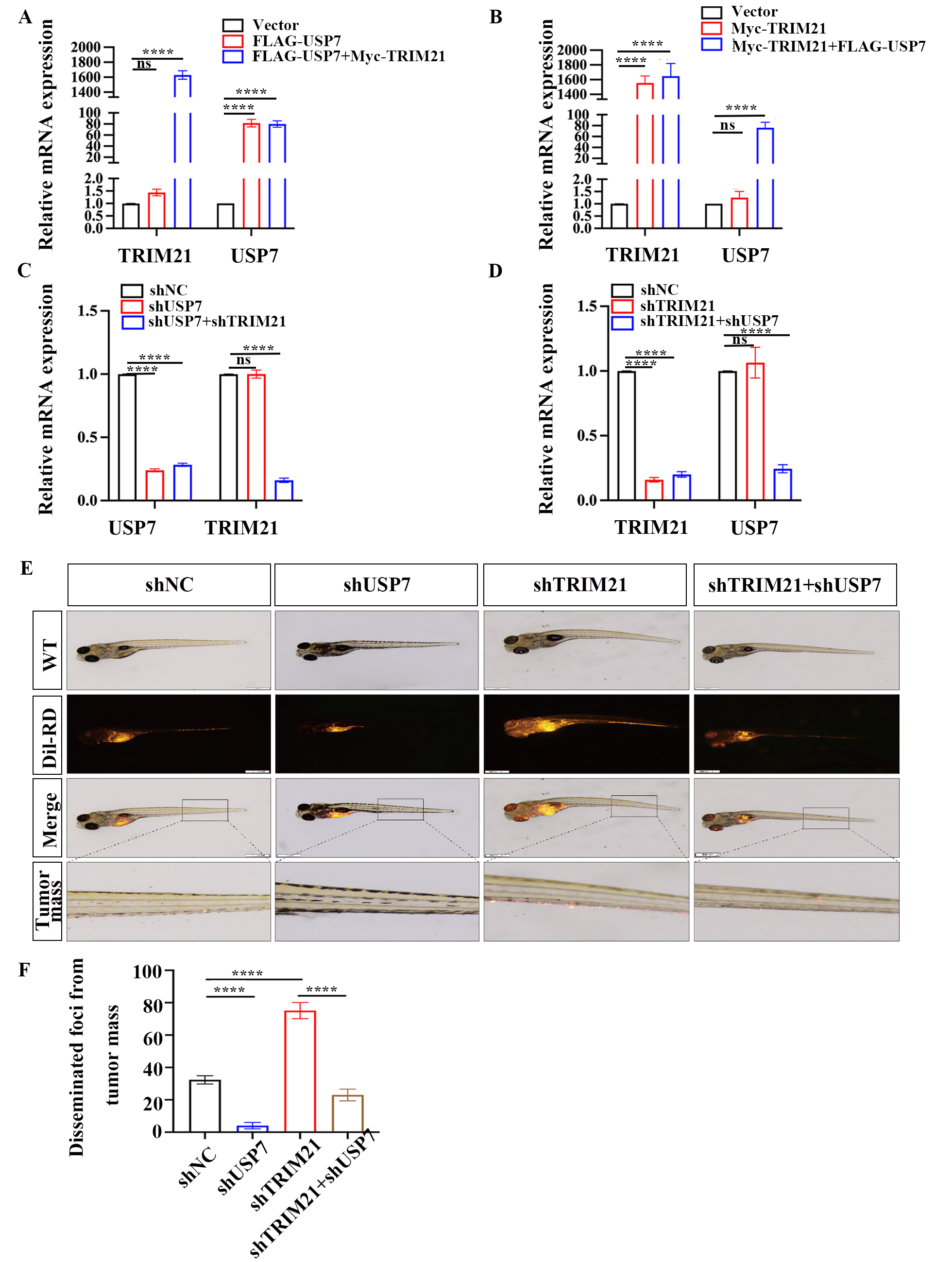

Supplement: Supplementary file 6 — Additional file 6: Supplemental Figure 6. TRIM21 can inhibit proliferation of RMS by regulating SNAI2 in vitro. A, B RT-PCR indicated that the mRNA expression levels of USP7 and TRIM21 in RD cells co-translated with USP7 and TRIM21 expressing plasmids. C, D The mRNA levels of USP7 and TRIM21 were analyzed by RT-PCR in RD Cells. E, F The zebrafish model was used to analyze the dissemination and metastasis of the four groups of RD cells (E) and the tumor mass was statistically analyzed (F).Data are presented as the Mean ± SD. ****P < 0.0001. [file 13046_2024_3056_MOESM6_ESM.png]
